# Integrated transcriptomic, proteomic and metabolomic analyses revealing the roles of amino acid and sucrose metabolism in augmenting drought tolerance in *Agropyron mongolicum*

**DOI:** 10.3389/fpls.2024.1515944

**Published:** 2024-12-16

**Authors:** Xiaoran Ma, Qingwei Liang, Yusi Han, Lu Fan, Dengxia Yi, Lin Ma, Jun Tang, Xuemin Wang

**Affiliations:** ^1^ Institute of Animal Sciences, Chinese Academy of Agricultural Sciences, Beijing, China; ^2^ Chifeng Institute of Agriculture and Animal Husbandry Science, Chifeng, China

**Keywords:** *Agropyron mongolicum*, drought stress, proteomics, transcriptomics, metabolomics

## Abstract

Drought, a major consequence of climate change, initiates molecular interactions among genes, proteins, and metabolites. *Agropyron mongolicum* a high-quality perennial grass species, exhibits robust drought resistance. However, the molecular mechanism underlying this resistance remaining largely unexplored. In this study, we performed an integrated analysis of the transcriptome, proteome, and metabolome of *A. mongolicum* under optimal and drought stress conditions. This combined analysis highlighted the pivotal role of transporters in responding to drought stress. Moreover, metabolite profiling indicated that arginine and proline metabolism, as well as the pentose phosphate pathway, are significantly involved in the drought response of *A. mongolicum*. Additionally, the integrated analysis suggested that proline metabolism and the pentose phosphate pathway are key elements of the drought resistance strategy in *A. mongolicum* plants. In summary, our research elucidates the drought adaptation mechanisms of *A. mongolicum* and identifies potential candidate genes for further study.

## 1 Introduction

In the context of agricultural development, drought serves as a pivotal constraint, hindering plant growth, diminishing crop yields, and limiting the extent of cultivable land. Arid and semi-arid regions, which represent about 36% of the global land area and 43% of the world’s arable land, are particularly susceptible to this issue. As a result, global water scarcity has emerged as a significant factor contributing to the decline in crop yields (Du et al., 2017; Han et al., 2017; Zurbriggen et al., 2010). To survive under such conditions, plants have developed a range of defense mechanisms that facilitate adaptation to arid environments. The study of these adaptive mechanisms is essential for advancements in agriculture, crop breeding, and the enhancement of varietal resilience (Alvarez et al., 2008).

Recent studies on the drought resistance of plant stems and roots have underscored the importance of several key agronomic traits. Firstly, the length of roots during the seedling stage is identified as a critical factor in improving crop yield under drought stress (Ahmed et al., 2019). Additionally, in situations where subsoil moisture is limited, enhancing root mass and density can significantly improve yield components by facilitating more efficient water uptake (Fang et al., 2017). Furthermore, the total root length is another trait that plays a role in drought resistance, as it affects the soil distribution of roots and the amount of water they can absorb (Wasaya et al., 2018). Moreover, the survival or vigor of seedlings is a practical indicator for assessing drought tolerance in controlled laboratory conditions. Lastly, early growth, or vigor, especially concerning the size of leaves and stems at the early stages of plant development, is a crucial crop trait associated with enhanced water utilization, greater biomass, and higher grain yields (Zhao et al., 2019).

*Agropyron mongolicum* Keng, a diploid allogamous species (2n = 2x = 14, PP), belongs to the genus *Agropyron Gaertn* and is renowned as a high-quality perennial forage grass. It is predominantly distributed across regions in China, such as Hebei, Shanxi, Gansu, Ningxia, and Inner Mongolia (Boyer, 1982). This species flourishes in sandy and desert grasslands and frequently acts as a companion species in areas prone to desertification (Vij and Tyagi, 2010). *A. mongolicum* is primarily cultivated in arid and semi-arid regions characterized by water scarcity, where drought stress markedly constrains its growth and productivity. In 1990, the species was officially designated as a wild-type cultivated variety by the National Forage Variety Appraisal Committee.

*Agropyron mongolicum* exhibits high seed-setting and germination rates, robust vitality and adaptability, early greening in spring, and late senescence in autumn. These attributes render it an ideal forage option for areas with scarce resources, especially during the winter and spring seasons. The species boasts a high nutritional content, tender shoots and roots, and is characterized by good palatability and resistance to cold, drought, adverse soil conditions, and sandstorms. These characteristics make *A. mongolicum* particularly suitable for enhancing natural grasslands, serving as a windbreak and for sand stabilization, as well as for establishing artificial grasslands. Despite the substantial research on its agronomic properties, there has been limited exploration into the molecular basis of its drought resistance. Considering its outstanding drought tolerance, *A. mongolicum* shows potential as a genetic resource for the development of stress-resistant crops through breeding programs (Che and Li, 2007; Esfahanian et al., 2017; Zurbriggen et al., 2010).

Drought stress markedly impacts the growth and productivity of *A. mongolicum*, triggering alterations in its intrinsic molecular responses. An integrated multi-omics strategy yields profound insights into essential biological processes, metabolic pathways, and regulatory networks within plants (Amiour et al., 2012; Bittencourt et al., 2022; Chin et al., 2020; Leao et al., 2022; Li et al., 2019; Moreno et al., 2021; Remmers et al., 2018; Shu et al., 2022; Srivastava et al., 2013; Wang et al., 2019; Yun et al., 2019). Advances in sequencing technologies have facilitated comprehensive analyses of gene expression patterns, providing robust tools for molecular diagnosis and classification (Xia et al., 2023).

Multi-omics technologies have been extensively employed to elucidate the mechanisms of stress resistance in diverse plants and crops, encompassing responses to water stress, salt stress, disease resistance, and drought stress. These studies have pinpointed genes and metabolites associated with pivotal traits, as well as variations in the accumulation of bioactive components. Findings indicate that various plants and crops mount unique responses to different abiotic stresses. For instance, multi-omics high-throughput technologies have uncovered that E3-ubiquitin ligase proteins play a role in regulating abiotic stress responses in rice (Li et al., 2023; Melo et al., 2021). In wheat, proteomic and metabolomic approaches have been utilized to investigate the drought resistance mechanisms of two spring wheat varieties, Bahar (drought-resistant) and Kavir (drought-sensitive). Metabolomic analysis revealed marked alterations in primary metabolites, including amino acids, sugars, and organic acids, under drought stress. Specifically, the Bahar variety exhibited accumulation of branched-chain amino acids, lysine, proline, aromatic amino acids, arginine, and methionine, with the branched-chain pathway involved in tryptophan accumulation activated, thus contributing to auxin production. Conversely, the Kavir variety’s metabolome exhibited fewer affected pathways, with purine metabolism being one of only two significantly impacted under stress conditions (Amirbakhtiar et al., 2019; Francki et al., 2016; Guo et al., 2020b; Michaletti et al., 2018; Saini et al., 2022; Shewry et al., 2017).

In the present study, we performed an integrated omics analysis of the transcriptome, proteome, and metabolome in the shoots and roots of *A. mongolicum* under both normal and drought stress conditions to elucidate its drought tolerance mechanisms. Differential expression analysis identified substantial changes in transcripts, proteins, and metabolites among various plant tissues in response to drought stress. Our comprehensive multi-omics investigation highlighted the pivotal roles of arginine and proline metabolism, as well as the pentose phosphate pathway, in the drought resistance of *A. mongolicum*. These findings offer new perspectives on the genes that could potentially enhance plant drought tolerance.

## 2 Materials and methods

### 2.1 Plant material

The *A. mongolicum* was gifted by Inner Mongolia Agricultural University. The seeds were initially surface-sterilized with 2% sodium hypochlorite for 45 minutes, then soaked overnight in distilled water. Germination took place on a moist blotting sheet within a petri dish at 28°C, under a 14-hour light/10-hour dark photoperiod. Seven days after germination, the seedlings were transferred to a hydroponic system with 1/2-strength Murashige and Skoog (MS) medium as the nutrient source, and were cultivated for an additional 14 days. The 14-day-old seedlings were subjected to a 20% (w/v) PEG 6000 solution to simulate drought stress. Following a 24-hour treatment period, both treated (drought-stressed) and untreated (control) seedlings were harvested, flash-frozen in liquid nitrogen, and stored at -80°C for subsequent analysis. The growth and sample collection were conducted across three independent biological replicates. These samples were utilized for subsequent transcriptomic, proteomic, and metabolomic analyses.

### 2.2 Transcriptome analysis

Total RNA was extracted using ethanol precipitation and the CTAB-PBIOZOL method. The quality and quantity of the RNA samples were evaluated using a Qubit fluorometer and a Qsep400 high-throughput bio-fragment analyzer (Met ware Biotechnology Co., Ltd. Wuhan, China). The library preparation followed standard Illumina protocols, and sequencing was carried out on the Illumina Nova-seq 6000 platform by a commercial service provider. The raw reads were filtered using Fastq to remove reads with adapters (Patel and Jain, 2012). Owing to the absence of a reference genome, transcriptome assembly was conducted *de novo*. Novel gene prediction was performed using String Tie, which applies network flow algorithms and optional *de novo* transcript assembly to splice transcripts. Gene expression was quantified using feature counts to calculate gene alignment statistics, followed by the computation of FPKM (Fragments Per Kilobase Million mapped reads) values based on gene length. Differential gene expression (log2 fold change ≥ +1 or ≤ -1 and FDR ≤ 0.05) was analyzed using edgeR (Robinson et al., 2010).

### 2.3 Protein sample preparation and proteomic analysis

The protein extraction and nano LC-MS/MS analysis were performed following the method described by (Singh et al., 2023). Total protein was extracted by suspending 1 g of ground tissue in an extraction buffer containing 1% SDS, 100 mM Tris-HCl, 7 M urea, 2 M thiourea, 1 mM PMSF, and 2 mM EDTA. The mixture was shaken, ultrasonicated on ice for 10 minutes, and the centrifuged to obtain the protein solution. Four times the volume of chilled acetone was added, and the proteins were precipitated overnight at -20°C, followed by centrifugation at 4°C to collect the precipitate. The precipitate was washed with cold acetone and dissolved in 8 M urea. Protein concentration was determined using a BCA kit according to the manufacturer’s instructions.

For tryptic digestion, equal number of proteins were taken from each sample. The proteins were reduced with 10 mM DTT for 45 minutes at 37°C and alkylated with 50 mM iodoacetamide (IAM) for 15 minutes in the dark at room temperature. The protein precipitate was collected using 4 times the volume of chilled acetone precipitation at -20°C for 2 hours and resuspended in 200 μL of 25 mM ammonium bicarbonate solution. Protein was digested overnight at 37°C with 3 μL of trypsin (Promega). After digestion, peptides were desalted using a C18 cartridge, drying using a vacuum concentrator, and redissolved in 0.1% (v/V) formic acid.

LC-MS/MS analysis was performed on an Orbitrap Astral MS system coupled to a Thermo Scientific™ Vanquish™ Neo UHPLC system, Samples were injected via an autosampler and trapped on a PepMap Neo Trap Cartridge column (300 μm ×5 mm, 5 μm), then separated on an Easy-Spray™ PepMap™ Neo UHPLC column (150 µm × 15 cm, 2 μm) over a 22-minute gradient. For DIA experiments, the Orbitrap Astral MS was set to a full MS resolution of 240,000 at 200 m/z with a scan range of 380-980 m/z. The full MS AGC was set to 500%. Fragment ion scans were recorded at a resolution of 80,000 and Maximum injection time (ms) of 3 ms using 299 windows of 2-Th scanning from 380-980 m/z. Ions were fragmented using HCD with a normalized collision energy (NCE) of 25%.

MS raw data were analyzed using DIA-NN (v1.8.1) with a library-free method. Three databases were used to create a spectra library utilizing deep learning algorithms from neural networks: the MWXS-24-694-a_*Agropyron_mongolicum*_Keng. blast. pep. fasta (containing 25,403 sequences)、MWXS-24-694-a_*Agropyron_mongolicum*_Keng.angel.pep.fasta (containing 337 sequences)、and iRT2.fasta (containing 1 sequences).of. The Match Between Runs (MBR) option was employed to generate the spectral library from the DIA data, which was then reanalyze using this library. The Flase discovery rate (FDR) for search results was set to < 1% at both the protein and precursor ion levels. The remaining identifications were used for further quantitative analysis. Differentially expressed proteins (with a log2 fold change ≥ +1 or ≤ -1 and FDR ≤ 0.05) were identified using edgeR.

### 2.4 Metabolite profiling using GC-MS

The samples were subjected to vacuum freeze-drying using a lyophilizer (Scientz-100F) and then ground into powder form using a grinder (MM 400, Retsch) at 30 Hz for 1.5 minutes. Fifty milligrams of powdered sample were weighed using an electronic balance (MS105Dμ), followed by the addition of and then 1200 μL of a -20°C pre-cooled 70% methanolic aqueous extract solution (adjusted to maintain the ratio of 1200 μL extract per 50 mg sample). internal standard was added, the mixture was vortexed every 30 minutes for 30 seconds, repeating this process 6 times. After centrifugating at 12,000 rpm for 3 minutes, the supernatant was collected, filtered through a 0.22 μm microporous membrane, and stored in injection vial for UPLC-MS/MS analysis. Metabolites levels were normalized using ribitol as an internal standard, and differential expression analysis (log2fold change ≥ +1 or ≤ - 1 and FDR ≤ 0.05) was perform educing edgeR (Robinson et al., 2010).

### 2.5 Integration of transcriptome, proteome and metabolome data

Based on the results of Kyoto Encyclopedia of Genes and Genomes (KEGG) enrichment analysis, pathways shared between differentially expressed genes (DEGs) and proteins (DEPs), DEGs and differentially expressed metabolites (DEMs), or DEPs and DEMs were selected to assess their roles in *A. mongolicum* under drought conditions.

## 3 Results

To elucidate the regulatory mechanisms of *A. mongolicum* under drought stress, we conducted transcriptomic, proteomic, and metabolomic analyses on drought-stressed seedlings. Drought stress negatively impacts multiple biological processes in plants, such as growth and physiological metabolism. Plants counteract drought stress by engaging physiological functions that mitigate, alleviate, or repair damage. Proline (Pro), a compound ubiquitous in plants, serves as a pivotal marker for evaluating drought tolerance, with elevated Pro levels being associated with enhanced drought resilience. Consequently, we conducted physiological assays to assess the drought response characteristics of *A. mongolicum* shoots. Our findings revealed a marked increase in proline activity 24 hours after the onset of drought stress (**Supplementary Figure 1**).

### 3.1 Transcriptional dynamics of *A. mongolicum* under drought stress

**Figure 1:**
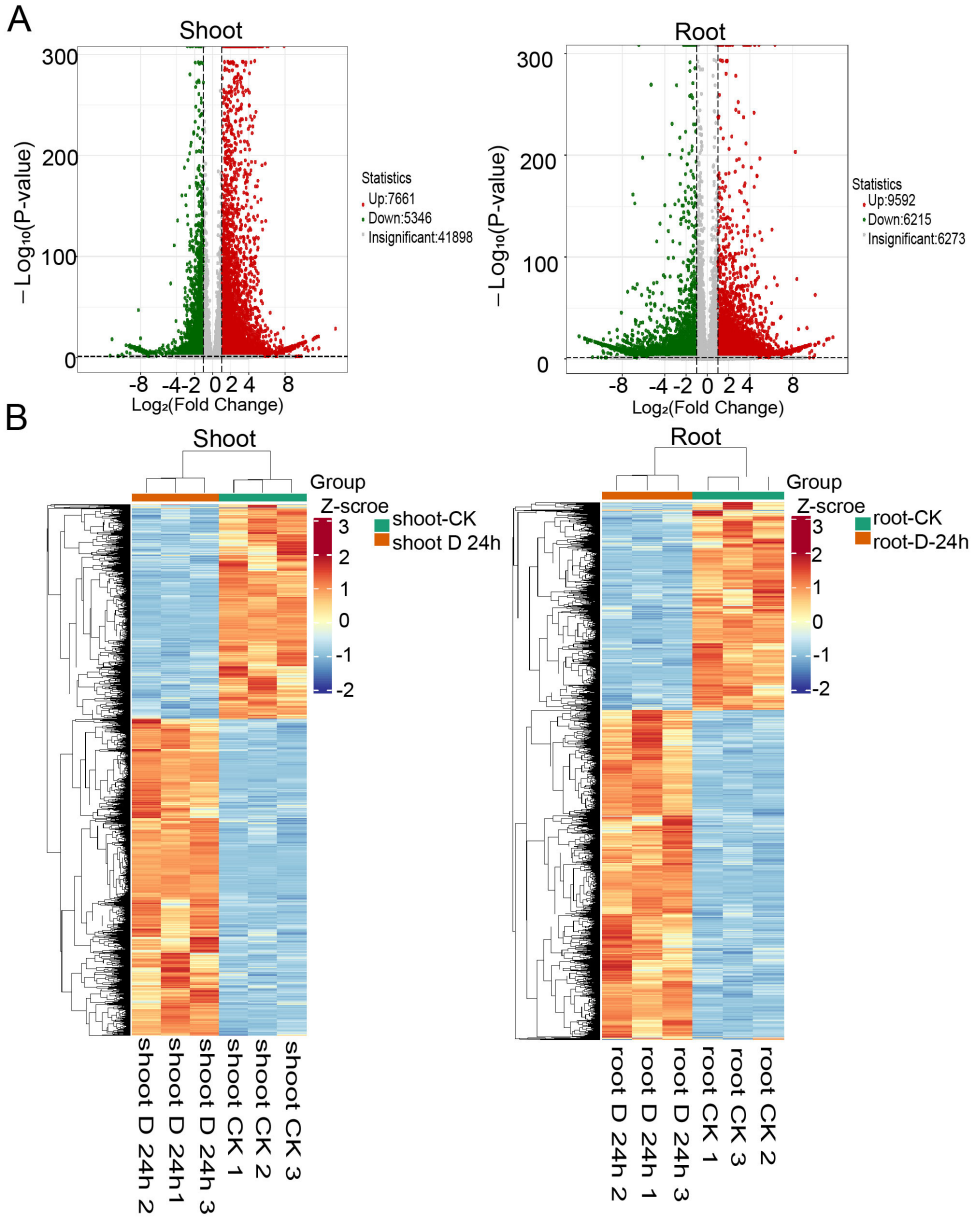
Differential expression genes identified under PEG treatment. **(A)** Venn diagrams showing the differentially expressed genes identified in PEG treatment in shoots (left) and roots (right). **(B)** Heatmaps displaying the normalized read counts of differentially expressed genes in PEG treatment in shoots (left) and roots (right).

To elucidate the transcriptional changes elicited by drought stress, RNA sequencing was performed on the roots and shoots of 14-day-old seedlings that were either treated or untreated with PEG6000 for 24 hours. A total of 104.24 Gb of clean data was generated from the 12 samples, with each sample exceeding 7 Gb and a Q30 score greater than 94% (**Supplementary Table 1**). The differential gene expression analysis, conducted using DESeq2, involved P-value adjustments according to the Benjamini & Hochberg method. Our analysis revealed 7661upregulated and 5346 downregulated genes in the shoots (fold change ≥2 and q-value ≤0.05), as well as 9,592 upregulated and 6,215 downregulated genes in the roots (**Figure 1A**, **Supplementary Tables 2**, **3**). Hierarchical clustering analysis uncovered distinct expression profiles between the shoots and roots. Furthermore, to assess the expression patterns of differentially expressed genes (DEGs) under varying treatment durations, we aggregated the DEGs from the comparison groups, performed hierarchical clustering, normalized the data using Z-score, and constructed a heatmap (**Figure 1B**). The heatmap revealed that the 0 h *vs* 24 h comparison groups exhibited opposite trends.

**Figure 2:**
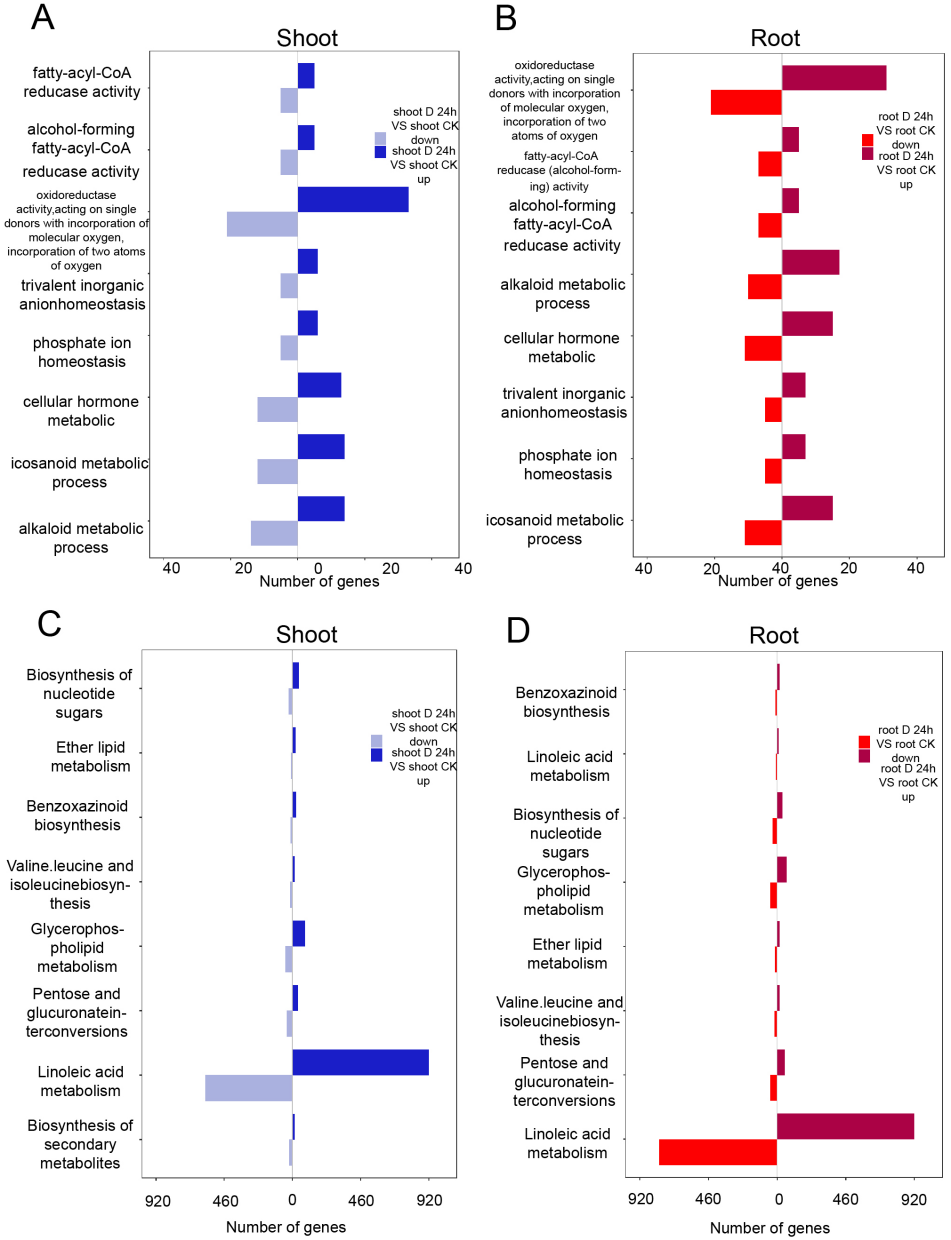
Differential expression genes identified under PEG treatment. **(A, B)** GO analysis of the differentially expressed genes in shoots **(A)** and roots **(B)**. **(C, D)** KEGG analysis of the differentially expressed genes in shoots **(C)** and roots **(D)**.

Gene Ontology (GO) enrichment analysis revealed that both upregulated and downregulated genes in the shoots were significantly associated with metabolic pathways, including those involved in redox enzyme activity, α-linolenic acid metabolism, and cytokinin metabolism (**Figure 2A**). Similarly, in the roots, pathways related to eicosanoid metabolism and cytokinin metabolism were prominently enriched (**Figure 2B**). Utilizing the Kyoto Encyclopedia of Genes and Genomes (KEGG) database, we identified key pathways that were enriched under drought stress conditions. Specifically, in the shoots, genes exhibiting changes in expression were primarily enriched in pathways such as secondary metabolite biosynthesis, glycerophospholipid metabolism, and pentose and glucuronate interconversions (**Figure 2C**). In the roots, the enriched pathways encompassed nucleotide sugar biosynthesis, glycerophospholipid metabolism, and pentose and glucuronate interconversions (**Figure 2D**).

**Figure 3:**
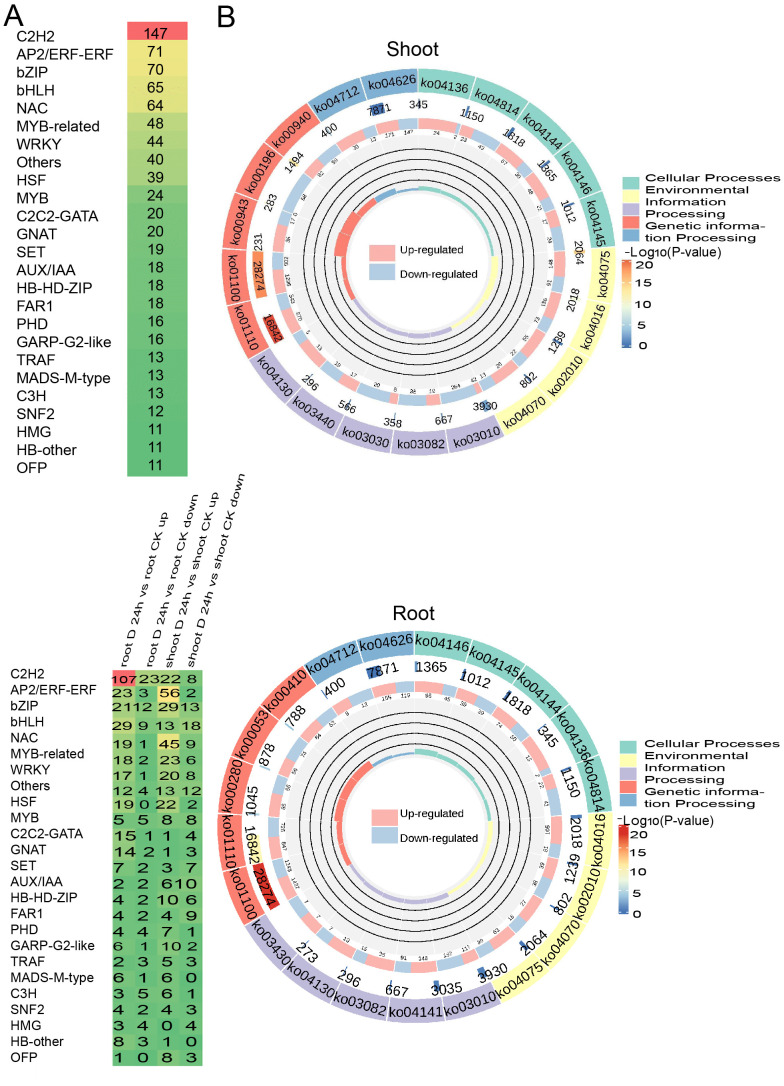
Differentially expressed transcription factors (DE-TFs) identified under PEG treatment. **(A)** The distribution of differentially expressed transcription factors (DE-TFs) under drought conditions in both shoot and root, with a family-wise breakdown of the up-regulated DE-TFs in response to drought stress. **(B)** KEGG Enrichment Circle Plot of Differential Genes expressed genes under drought conditions in the shoot (above) and root (below).

In a similar vein, we identified 841 differentially expressed transcription factors (TFs) belonging to 26 TF families under drought conditions. Notably, TF families such as *C2H2*, *AP2/ERF*, *bZIP*, *bHLH*, and *NAC* were highly expressed in both shoots and roots, highlighting their pivotal roles in regulating the plant’s response to drought stress (**Figure 3A**). Moreover, the KEGG enrichment circle plot analysis revealed that differentially expressed genes (DEGs) in both shoot and root tissues were primarily enriched in pathways including KEGG pathway ko1100 (Metabolic pathways), ko01110 (Biosynthesis of secondary metabolites), ko04626 (Plant-pathogen interaction), and ko03010 (Ribosome), suggesting a significant association between amino acid metabolism and oxidative phosphorylation (**Figure 3B**). The comprehensive analysis indicates that DEGs are particularly enriched in amino acid biosynthesis and carbohydrate metabolism pathways.

### 3.2 Proteomic analysis of *A. mongolicum* in response to drought stress

**Figure 4:**
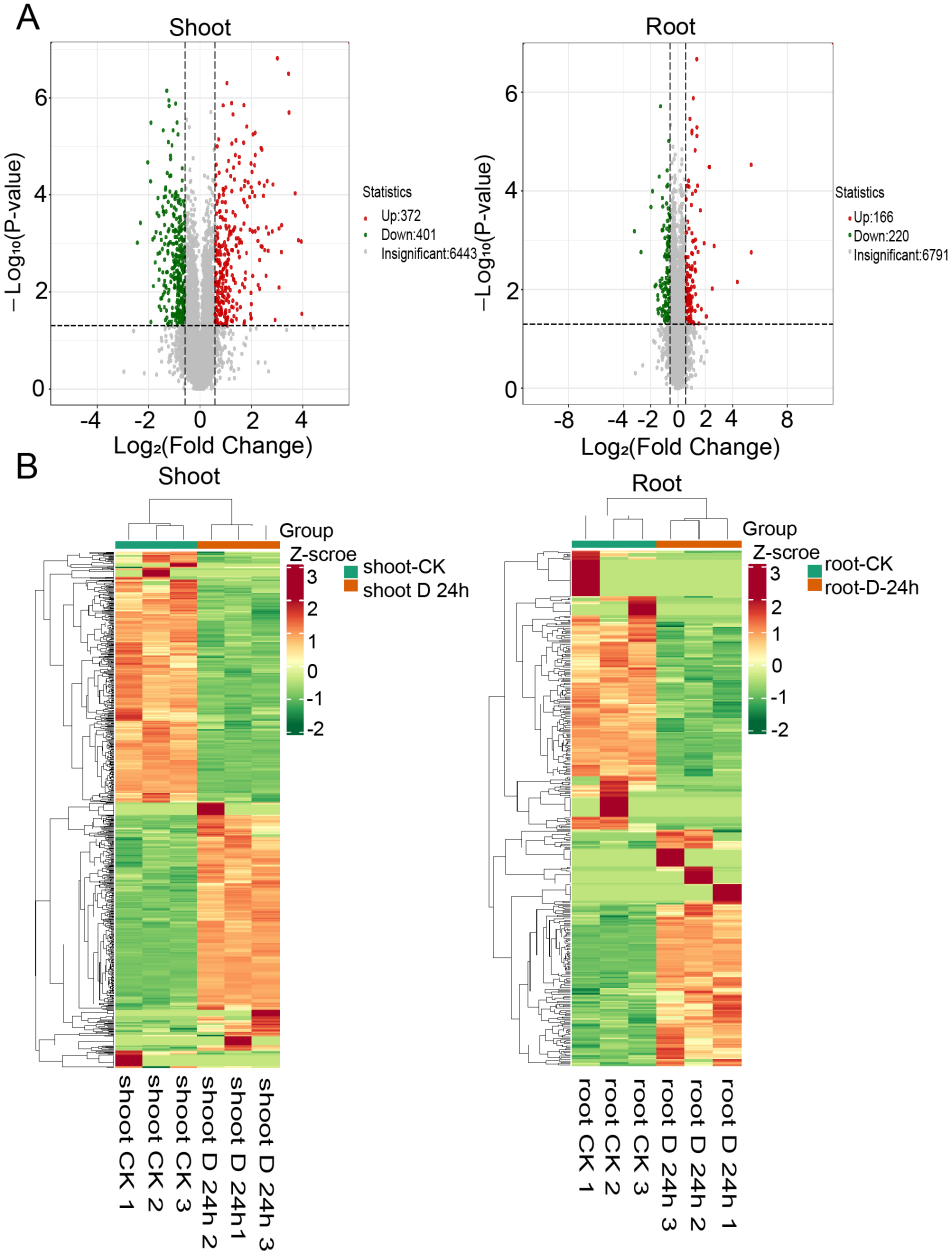
Proteome response of shoot and root under drought stress conditions. **(A)** Volcano plot of differentially expressed proteins (log2 fold change≥1 or ≤1 and P-value ≤ 0.05) under drought (DS) and control (CK) conditions in shoots (left) and roots (right) **(B)** Heatmaps displaying the normalized read counts of differentially expressed proteins in PEG treatment in shoots (left) and roots (right).

**Figure 5:**
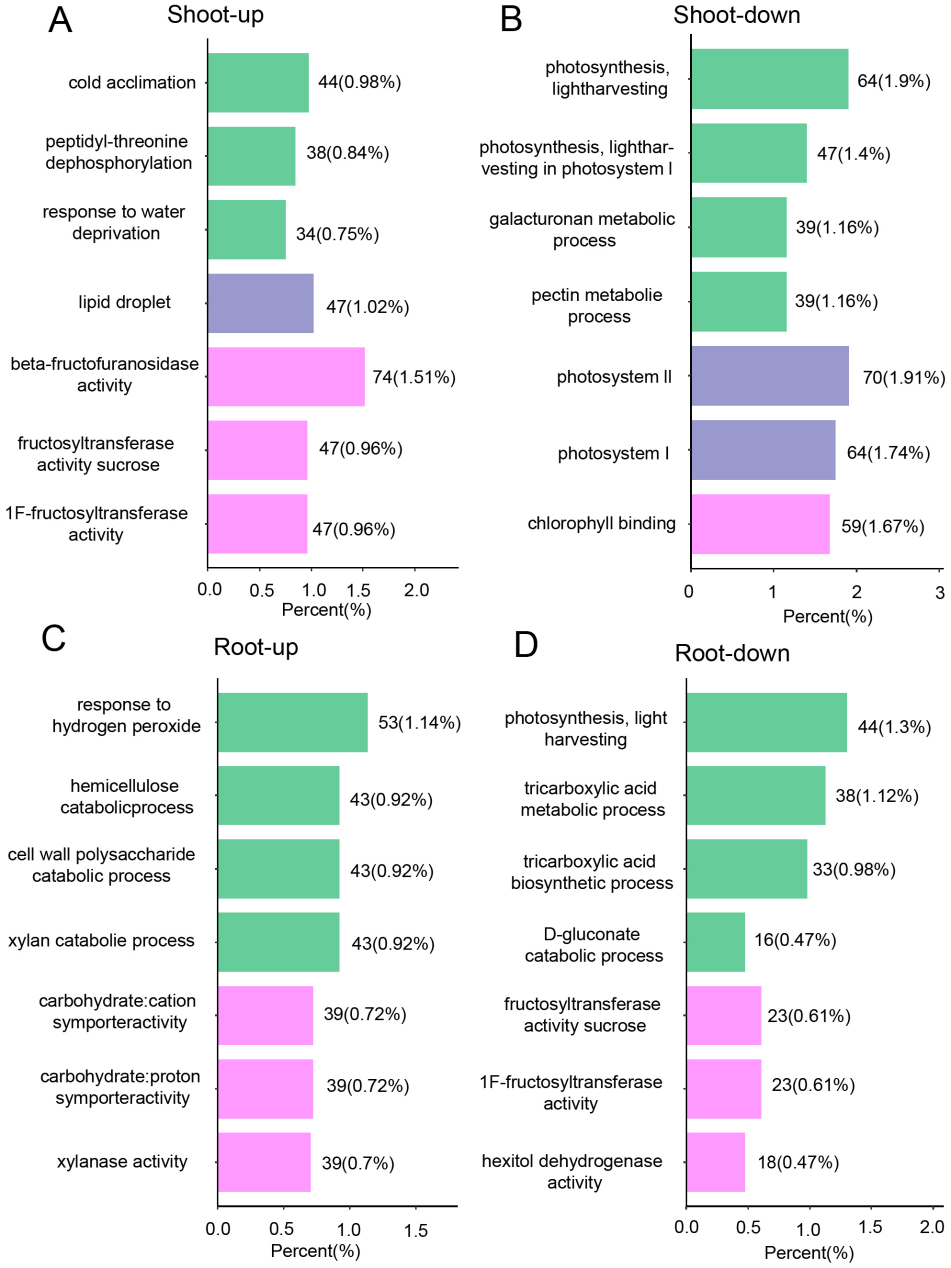
Proteome response of shoot and root under drought stress conditions. GO analysis of differentially expressed genes that are up-regulated **(A)** and down-regulated **(B)** in shoot, as well as those that are up-regulated **(C)** and down-regulated **(D)** in root. The green purple, and pink terms present biological process (BP), cellular component, and the molecular function (MF) category, according to GO analysis, respectively.

To augment the insights gained from the global transcriptome analysis, we undertook a high-throughput proteomic study. This effort yielded a dataset comprising 95,877 peptides, which facilitated the identification and quantification of 7,286 distinct proteins (**Supplementary Figure 2**). Within this dataset, we identified 1,179 differentially expressed proteins (DEPs), with 773 in the shoots and 406 in the roots, based on a stringent criterion of a fold change ≥2 and a q-value <0.05 (**Figure 4A**). Hierarchical clustering analyses elucidated the differential protein expression patterns between shoots and roots, revealing distinct protein profiles for each tissue (**Figure 4B**). Gene Ontology (GO) enrichment analysis indicated that, under drought stress conditions, the shoots exhibited upregulated pathways such as cold acclimation, positive regulation of the response to water deprivation, and peptidyl-threonine dephosphorylation (**Figure 5A**). Conversely, downregulated pathways in the shoots pertained to photosynthesis, light harvesting in photosystem I, and the metabolic processes of galacturonan and pectin (**Figure 5B**). In roots, upregulated pathways were associated with responses to hydrogen peroxide, as well as the degradation of cell wall polysaccharides and hemicellulose (**Figure 5C**). In contrast, downregulated pathways in the roots involved activities such as fructose-bisphosphate aldolase, sucrose: sucrose 1F-fructosyltransferase, and hexose alcohol dehydrogenase (**Figure 5D**). These observations suggest that carbohydrate metabolism is profoundly impacted by drought stress, with alterations in both shoot and root tissues (**Figures 5A–D**).

**Figure 6:**
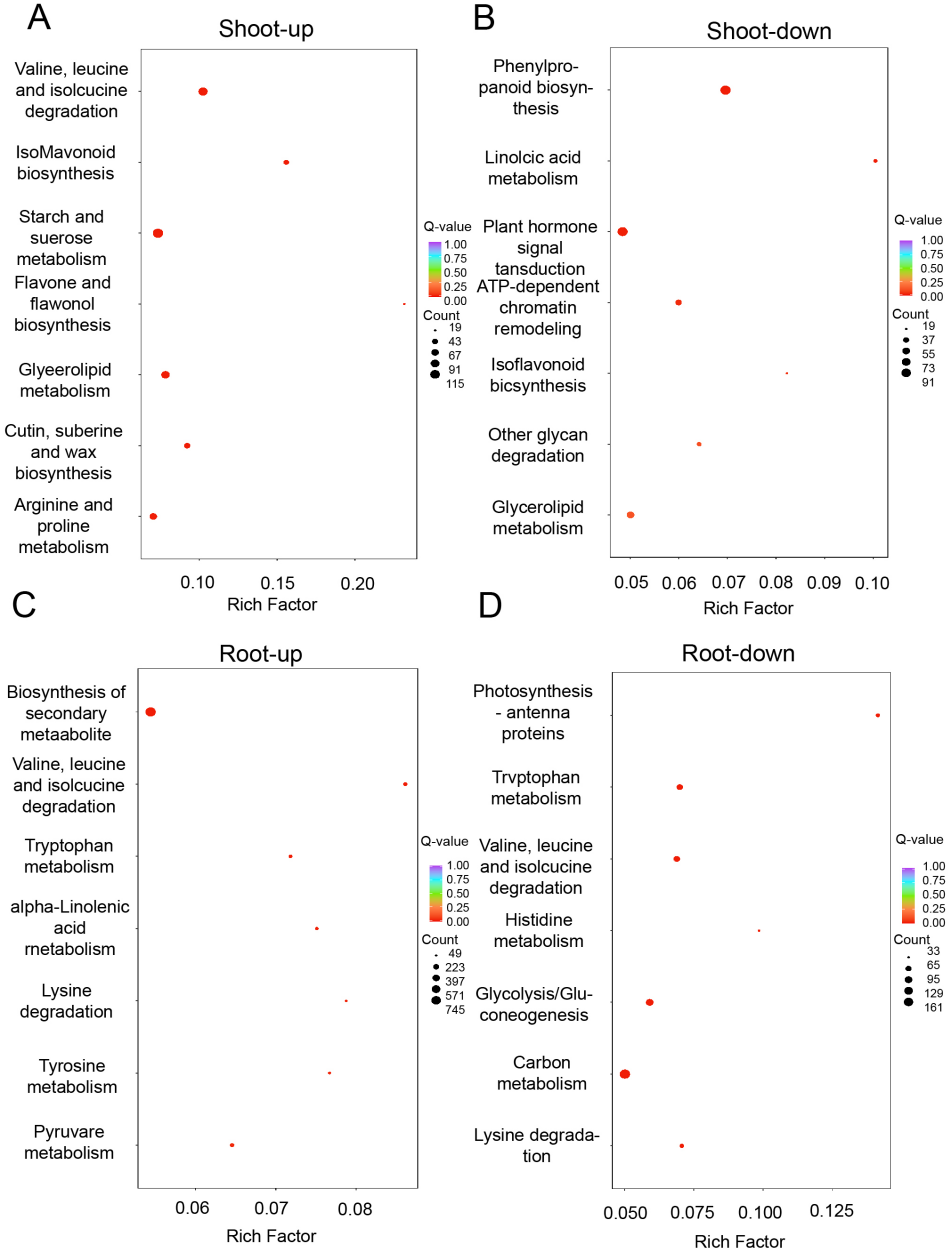
Proteome response of shoot and root under drought stress conditions. KEGG analysis of differentially expressed genes that are up-regulated **(A)** and down-regulated **(B)** in shoot, as well as those that are up-regulated **(C)** and down-regulated **(D)** in root.

To delve into the interplay between differentially expressed proteins (DEPs), amino acid biosynthesis, and carbohydrate metabolism, we conducted a Kyoto Encyclopedia of Genes and Genomes (KEGG) pathway analysis. In the shoots, the upregulated DEPs were predominantly enriched in pathways such as isoflavonoid biosynthesis, arginine and proline metabolism, degradation of valine, leucine, and isoleucine, as well as starch and sucrose metabolism (**Figure 6A**). Conversely, the downregulated DEPs pertained to pathways including phenylpropanoid biosynthesis, plant hormone signal transduction, and ATP-dependent chromatin remodeling (**Figure 6B**). In the roots, the upregulated DEPs were implicated in the degradation of valine, leucine, isoleucine, tryptophan, and lysine (**Figure 6C**), while the downregulated DEPs were associated with pathways such as photosynthesis-antenna proteins, valine, leucine, and isoleucine degradation, histidine metabolism, glycolysis/gluconeogenesis, and carbon metabolism (**Figure 6D**). The analysis highlights a significant enrichment of DEPs in the pathways of amino acid metabolism and carbohydrate metabolism, suggesting a crucial role for these pathways in the plant’s response to drought stress.

### 3.3 Integrated analysis of DEGs and DEPs

**Figure 7:**
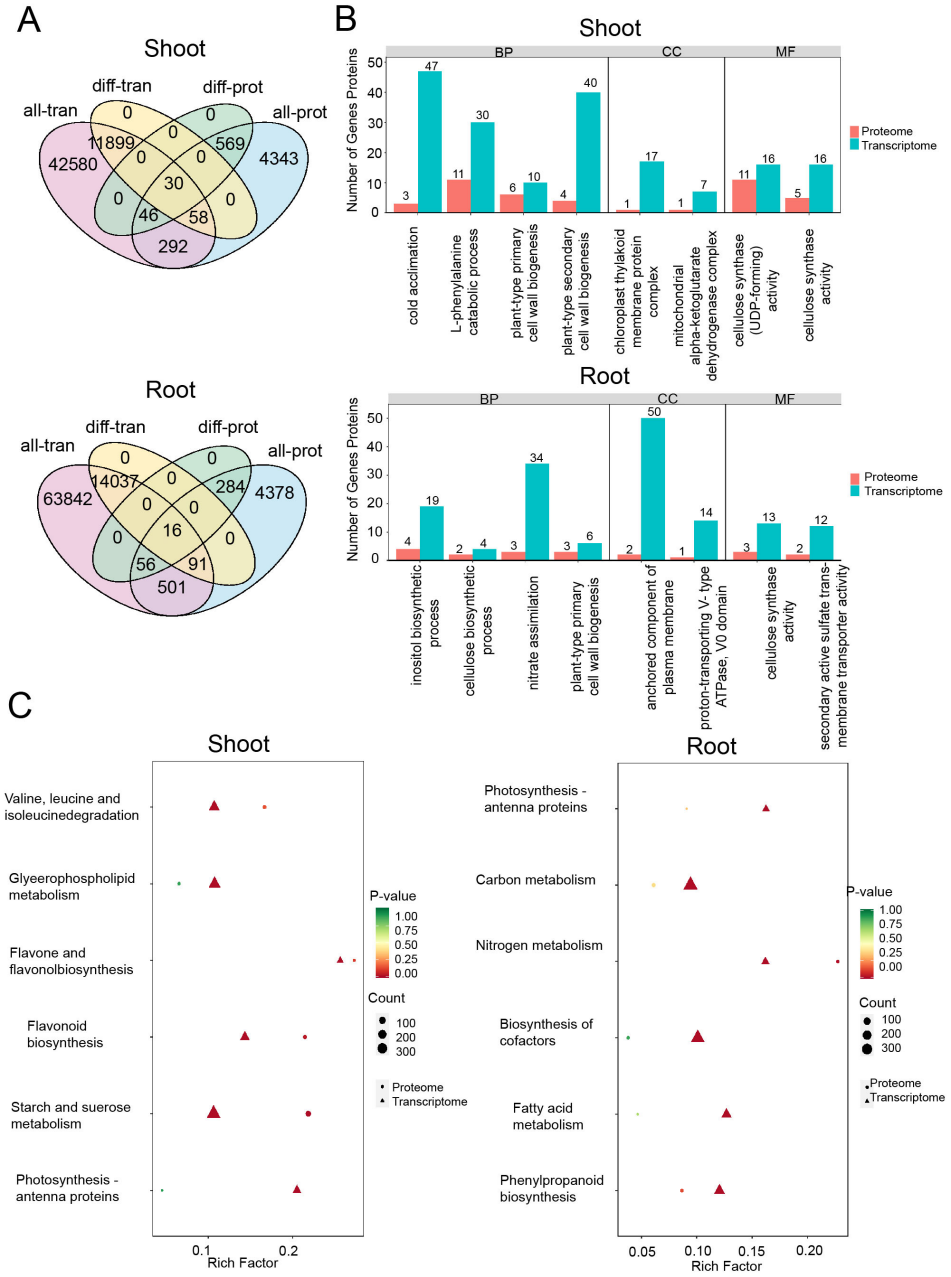
DEGs/DEPs identified in drought conditions. **(A)** Correlated genes and proteins expressed in drought conditions. all_ proteins/transcripts present the numbers of total proteins/transcripts identified under PEG treatments. diff_ proteins/transcripts present the numbers of differentially expressed proteins/transcripts identified in the treatments. **(B)** GO analysis of the differentially expressed genes in shoots (above) and roots (below). **(C)** KEGG analysis of the differentially expressed genes in shoots (left) and roots (right).

To explore the relationship between transcriptomic and proteomic responses to drought stress, we combined mRNA expression data from the transcriptome with protein abundance data from the proteome (**Figure 7A**). A comparative Gene Ontology (GO) enrichment analysis of differentially expressed genes (DEGs) and differentially expressed proteins (DEPs) uncovered critical biological pathways affected by drought stress. In the shoots, both DEGs and DEPs exhibited enrichment in pathways related to the catabolic process of L-phenylalanine, cold acclimation, and the biogenesis of plant-type primary and secondary cell walls. For cellular components (CC), an enrichment in the chloroplast thylakoid membrane was observed, and in terms of molecular functions (MF), the analysis emphasized the pathways involving cellulose synthase (UDP-forming) and cellulose synthase activity.

In roots, both differentially expressed genes (DEGs) and differentially expressed proteins (DEPs) were found to be enriched in biological processes (BP) such as cellulose biosynthesis and the plant-type primary cell wall pathway. The cellular component (CC) analysis highlighted the plasma membrane-anchored component pathway, while the molecular function (MF) analysis showed enrichment in pathways related to secondary active sulfate transporter activity. These findings imply that the identified DEGs and DEPs may be involved in regulating transport proteins, cell wall synthesis, and the production of metabolic products (**Figure 7B**). Notably, under drought stress, there was significant enrichment of pathways such as cold acclimation, plant-type primary cell wall process, secondary cell wall biogenesis, and nitrate assimilation in both DEGs and DEPs, emphasizing the significance of carbohydrate and amino acid metabolism in the plant’s response to drought.

Further Kyoto Encyclopedia of Genes and Genomes (KEGG) analysis of DEPs and DEGs indicated that in the shoots, the primary enrichment occurred in pathways related to flavonoid and flavanol biosynthesis, starch and sucrose metabolism, glycerophospholipid metabolism, and the photosynthesis-antenna proteins pathway. In contrast, the roots showed enrichment in pathways such as photosynthesis-antenna proteins, carbon metabolism, nitrogen metabolism, cofactor biosynthesis, fatty acid metabolism, and phenylpropanoid biosynthesis. These results underscore the importance of starch and sucrose metabolism, carbon metabolism, nitrogen metabolism, and phenylpropanoid biosynthesis in the plant’s response to drought stress, with a notable differential expression of genes and proteins that are integral to carbohydrate metabolism and amino acid metabolism (**Figure 7C**).

### 3.4 Metabolite analysis under drought stress

**Figure 8:**
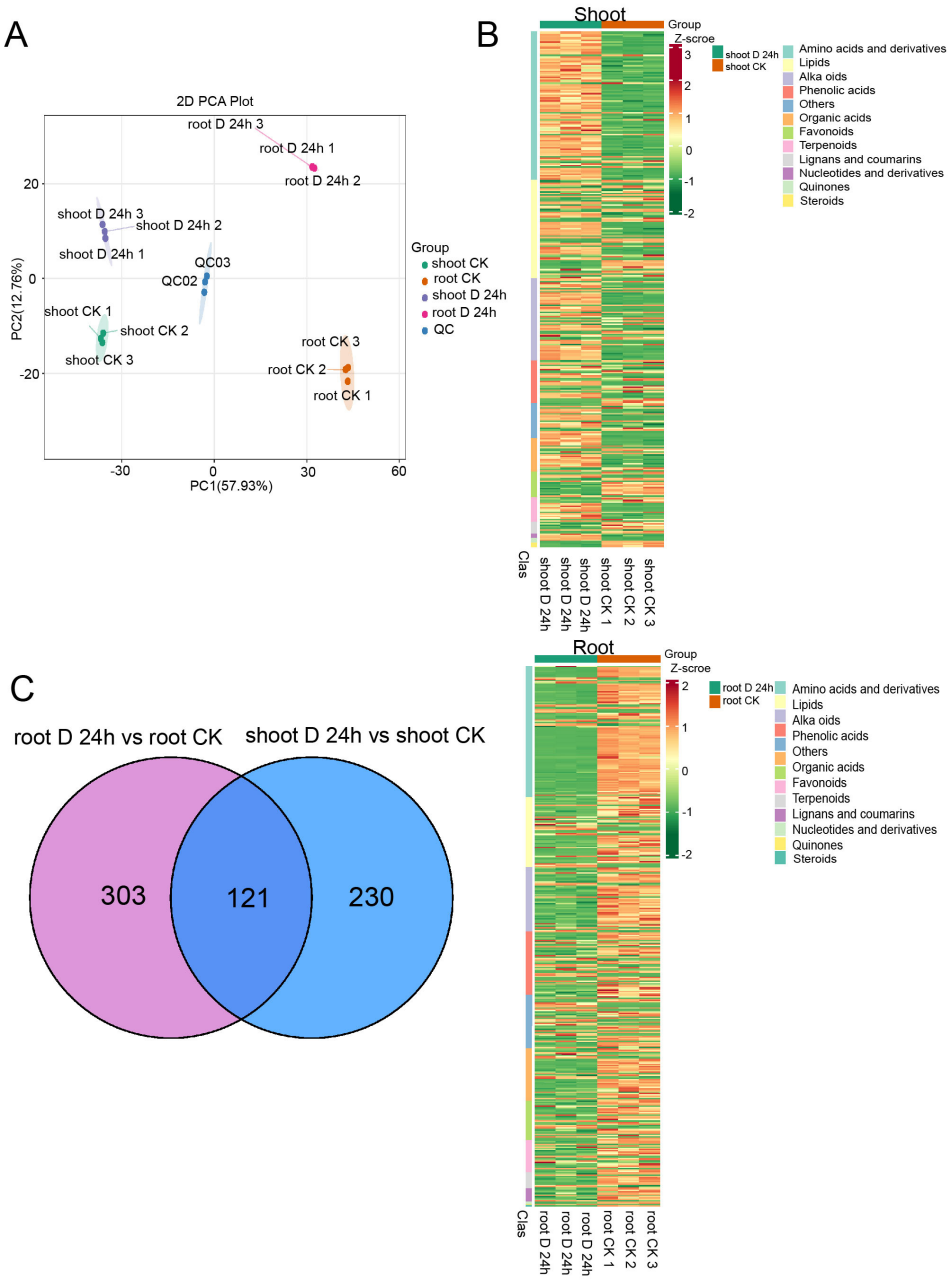
Metabolite response of shoot and root under drought stress conditions. **(A)** Principal components analysis (PCA) of the differentially expressed metabolites. **(B)** Heatmaps displaying the normalized read counts of differentially expressed proteins in PEG treatment in shoots (above) and roots (below). **(C)** Venn diagrams showing the differentially expressed metabolites identified in PEG treatment in shoots (right) and roots (left).

**Figure 9:**
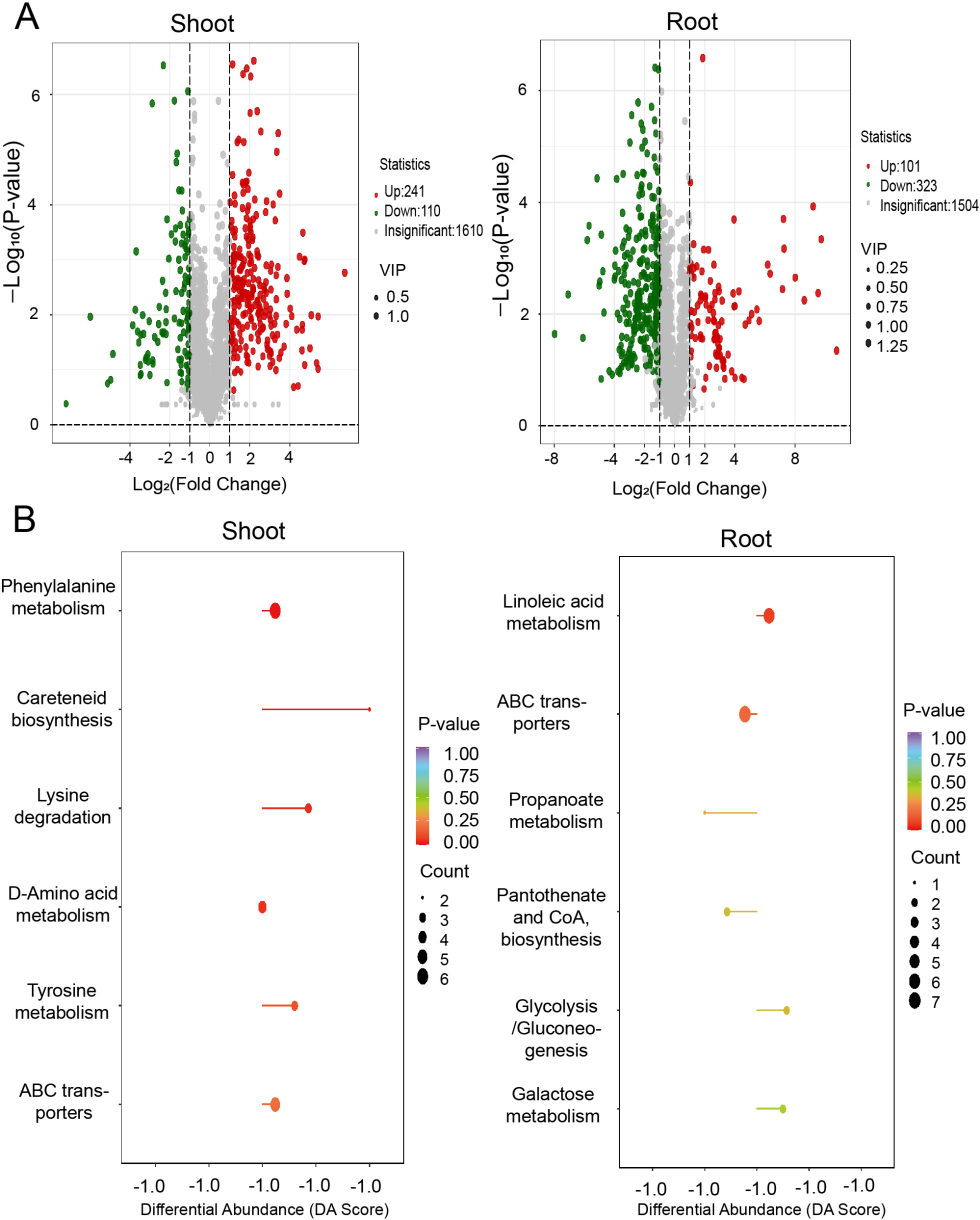
Metabolite response of shoot and root under drought stress conditions. **(A)** Venn diagrams showing the differentially expressed metabolites identified in PEG treatment in shoots (left) and roots (right). **(B)** KEGG analysis of the differentially expressed metabolites in shoots (left) and roots (right).

To connect transcriptomic and proteomic findings with metabolic pathways, we investigated the changes of major metabolites accumulation in shoots and roots of *A. mongolicum* under drought conditions. Employing ultra-high-performance liquid chromatography-tandem mass spectrometry (UPLC-MS/MS), we generated detailed metabolite profiles. Principal component analysis (PCA) revealed that the expression profiles of differentially expressed metabolites (DEMs) were distinct across the samples, with the exception of the shoot-CK and root-CK samples (**Figure 8A**). The shoot-D-24h and root-D-24h samples were distinctly separated from the quality control (QC) group based on principal component 1 (PC1), which accounted for 57.93% of the variance. A heatmap depicted the differential regulation of various metabolite categories under drought conditions (**Figure 8B**). Furthermore, a venn diagram showed that out of the DEMs, 121 were shared between shoots and roots, with 230 unique to shoots and 303 unique to roots (**Figure 8C**). The volcano plot in illustrated that there were 241 upregulated DEMs and 110 downregulated DEMs in the shoots, whereas in the roots, there were 110 upregulated DEMs and 323 downregulated DEMs (**Figure 9A**). KEGG pathway enrichment analysis showed that the upregulated DEMs in shoots were involved in pathways such as phenylalanine metabolism, amino acid biosynthesis, ABC transporters, and carotenoid biosynthesis. In roots, upregulated DEMs were linked to glycolysis/gluconeogenesis, galactose metabolism and linoleic acid metabolism, while downregulated DEMs were associated with propanoate metabolism, ABC transporters, and pantothenate and CoA biosynthesis (**Figure 9B**). After analyzing samples from CK and drought-treated groups (Shoot-D-24h and Root-D-24h), significantly differential metabolites were identified. In the CK and Shoot-D-24h samples, major upregulated metabolites included lipids (e.g., Beta-Hydroxypalmitic Acid), amino acids and derivatives (e.g., asn-pro-lys, aspl-pro, leu-Pro), and alkaloids (e.g., demissine, 6-Methylnicotinamide, alanine betaine) (**Supplementary Table 4**). For the CK and Root-D-24h samples, significantly downregulated metabolites included phenolic acids (e.g., 5-O-Galloyl-D-hamamelose*, 2-O-P-Coumaroylhydroxycitric Acid, 1,2-O-Diferuloylglycerol*), flavonoids (e.g., Tricetin 3’-glucuronide*, Tricin-7-O-(2’’-Malonyl) rhamnoside) and others (e.g., 4-Hydroxybenzaldehyde, Sorbitol-6-phosphate, Maltotriose) (**Supplementary Table 5**). Our findings suggested that metabolites in shoots were largely associated with amino acid metabolism, whereas those in roots were primarily linked to carbohydrate metabolism. Overall, the results indicate that metabolites are primarily associated with carbohydrate metabolism, amino acid metabolism.

### 3.5 Integrated analysis of transcriptomics, proteomics, and metabolomics

To gain a deeper understanding of the interplay among transcriptomics, proteomics, and metabolomics datasets, an integrated multi-omics analysis was conducted using a functional relatedness framework. The differentially expressed genes (DEGs, 26,131), differentially expressed proteins (DEPs, 1,179), and differentially expressed metabolites (DEMs, 775) identified in both shoots and roots were concurrently mapped to Kyoto Encyclopedia of Genes and Genomes (KEGG) pathways (**Supplementary Tables 6**–**8**), which allowed for the elucidation of their functional interconnections.

**Figure 10:**
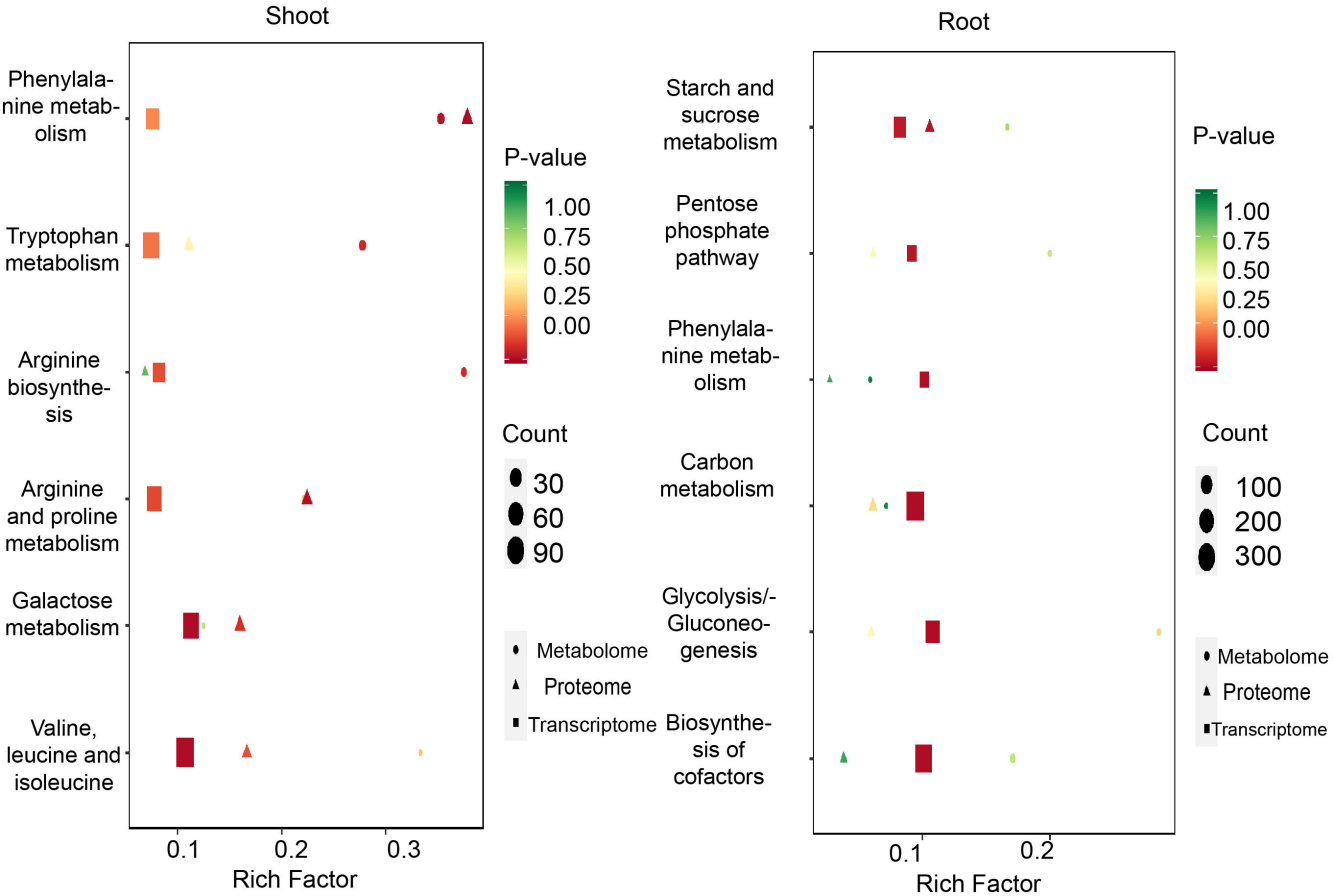
DEGs, DEPs and DEMs identified in drought conditions. KEGG analysis of the DEGs、DEPs and DEMs in shoots (left) and roots (right).

A KEGG pathway enrichment analysis was performed, and the bubble chart revealed that in the shoots, the most prominently enriched pathways included the degradation of valine, leucine, and isoleucine, galactose metabolism, arginine and proline metabolism, arginine biosynthesis, tryptophan metabolism, and phenylalanine metabolism. In contrast, the roots exhibited significant enrichment in pathways such as starch and sucrose metabolism, the pentose phosphate pathway, phenylalanine metabolism, carbon metabolism, and glycolysis/gluconeogenesis. Specifically, the interplay among genes, proteins, and metabolites was analyzed for the arginine and proline metabolism pathway and the pentose phosphate pathway (**Figure 10**). The findings suggested that under drought conditions, amino acid metabolism is predominantly enriched in the shoots, whereas carbohydrate metabolism is mainly enriched in the roots.

Moreover, the KEGG pathway maps illustrated that the arginine and proline metabolism pathway showed significant differences in the expression of related genes and proteins, including L-Arginine-P, Agmatine, Putrescine, and S-Adenosyl-L-methionine. In response to drought stress, the pentose phosphate pathway exhibited considerable changes in metabolite expression, with notable alterations observed in metabolites such as β-D-Fructose-1,6-bisphosphate, D-Erythrose 4-phosphate, D-Ribose 5-phosphate, and D-Glucose 6-phosphate (**Supplementary Figure 3**).

### 3.6 Differential genes under drought stress

This study employs integrated transcriptomic, proteomic, and metabolomic analysis techniques to systematically investigate the regulatory pathways of *A. mongolicum* under drought stress. Initially, in the proteomic screening, the GO database was analyzed for biological processes, cellular components, and molecular functions, identifying 16 highly expressed differentially expressed proteins (DEPs) associated with drought resistance. By querying the TAIR (https://www.arabidopsis.org/) website, the homologous gene families for these proteins were identified, including 14779 (*ASNS3*), 18687 (*ALDH7*), 18892 (*ALDH7B4*), 19487 (*AtUGT85A2*), 20718 (*TA*), 22603 (*SLC52A*), 17999 (*UDP-glucose*), 23189 (*AtXTH13*), 25241 (*AtOEP16-2*), 25473 (*GAPDH*), 25717 (*MBF1*), 994 (*ABCB*), 2543 (*LKR*), 3137 (*P53*), 8113 (*P5CS1*), and 17705 (*AtCIMS*). Following a combined analysis of transcriptomic, proteomic, and metabolomic data, four genes were selected: *ALDH7B4*, *ASNS3*, *P5CS1*, and *LKR*. The study revealed that each of these genes has corresponding differentially expressed genes (DEGs) and differentially expressed metabolites (DEMs). To validate the RNA-seq results for *ASNS3, P5CS1, LKR*, and *ALDH7B4* under drought stress, we performed an expression validation analysis using qRT-PCR. The results indicated that *ASNS3* expression was significantly upregulated at 24 h post-drought treatment relative to the control, while the expression levels of *P5CS1, LKR*, and *ALDH7B4* remained unchanged (**Supplementary Figure 4**). The KEGG Orthology (KO) numbers associated with these genes are Ko00280, Ko00310, Ko00330, Ko00380, and Ko01230 (**Supplementary Figure 5** and **Supplementary Table 9**). Additionally, the study discovered that *ASNS3*, *P5CS1*, and *LKR* are primarily enriched in amino acid biosynthesis pathways and their derivatives, whereas *ALDH7B4* is mainly associated with the pentose phosphate pathway.

## 4 Discussion

Drought is a major environmental stressor affecting plant growth. Therefore, elucidating the mechanisms of drought stress tolerance in *A. mongolicum* is important to enhance plant stress resistance. Multi-omics analysis has become established as an important analytical tool for understanding biochemical processes. The variations in amino acids profiles under distinct water stress conditions mirrored those found in *Lotus japonicus* (Sanchez et al., 2012) and maize plants (Alvarez et al., 2008). Proline levels are associated with severe water shortages in numerous plant species (Witt et al., 2012), with variations being genotype-specific and contingent upon the degree of water stress (Bowne et al., 2012). As a recognized compatible solute, prolines crucial for osmotic adjustments; It shields cellular structures during water stress periods and plays a significant role in neutralizing reactive oxygen species (ROS),thereby mitigating the negative impacts of drought stress on plant metabolism (Zadehbagheri et al., 2014).

### 4.1 Arginine and proline as determinative amino acids in drought stress

Amino acids are pivotal in modulating plant stress resilience to stress by affecting osmotic balance, ion movement, stomatal regulation, and chemical equilibrium. Proline, known for its role as an osmotic regulator, can enhances plant tolerance and defend cells against various abiotic stresses (Jia et al., 2016). In addition to functioning as an effective Osmo protectant, proline is involved in multiple biochemical processes, especially under challenging environmental conditions. It also plays essential roles in neutralizing free radicals, regulating cellular redox status, sequestering metal ions, and triggering protective responses within plants (Raza et al., 2023). Proline is synthesized from glutamate, a precursor, by the enzymes pyrroline-5-carboxylate synthase (*P5CS*) and pyrroline-5-carboxylate reductase (*P5CR*) (Hu et al., 1992). Inducing *P5CS* expression to elevate proline concentrations enables *Oryza sativa* to tolerate increased salt and water stress (Su and Wu, 2004). While arginine, an essential amino acid, serves diverse function in plants, providing crucial nitrogen and supporting cellular processes that enhance resistance to various abiotic stresses (Wang et al., 2024). Arginine has been demonstrated to stimulate the synthesis of proline (Bokhary et al., 2020).

Recent studies have highlighted the roles of urea cycle intermediates, including ornithine, aspartate, arginine, and citrulline, in the mechanisms that confer plant tolerance to abiotic stress (Blume et al., 2019; Kalamaki et al., 2009; Shi et al., 2013; Song et al., 2020). In peanut plants, aspartate, a precursor to arginine, also showed a decrease under most abiotic stress conditions. The significance of urea cycle intermediates, including ornithine, aspartate, arginine and citrulline, in enhancing plant resilience to abiotic stress has been increasingly recognized, leading to increased production of other amino acids (Matysiak et al., 2020; Song et al., 2020). Exogenous application of arginine has been shown to increase both fresh and dry weight of shoots and roots in maize plants, and pre-treatment with arginine has similarly effect on stressed sunflower plants. The stimulation effect of arginine on water deficit-stressed plants may be attributed to its role as an essential amino acid that promotes plant growth (Faraji and Sepehri, 2019). Arginine is crucial for plant growth and stress resistance. In some barley varieties, arginine can significantly enhance the morphological growth characteristics. Its application to *Helianthus annuus* L. results in increased branch and root length, as well as a greater number of leaves. Furthermore, pre-treatment with arginine can mitigate the effects of drought on wheat plant growth. Under drought stress, arginine treatment can lead to increased height, tiller number, leaves number, and flag leaf area in barley plants. These effects may be attributed to the conversion of L-arginine to proline and nitric oxide, and crucial for plants to counteract drought stress (Jarzyniak and Jasinski, 2014; Ramadan et al., 2019).

Drought stress triggers hyperosmotic stress in plant cells, negatively impacting growth, development, and productivity (Zhang et al., 2022). To counteract these effects, plants have various mechanisms such as enhancing photosynthesis and water use efficiency, or stimulating the synthesis and accumulation of osmoprotectant small molecules and antioxidant enzymes. Increased proline or soluble sugar accumulation under stress conditions can help maintain cellular osmotic potential (Sun et al., 2003).

After joint analysis, the genes regulated by amino acid and pentose phosphate metabolic pathways include *ASNS3, P5CS1*, and *LKY*, among which *ASNS3* has the highest expression abundance. Therefore, further studying the role of *Asn* gene in plant abiotic stress response is of great significance for improving plant stress tolerance and *sustainable agricultural production.*es are vital for plant adaptation to abiotic stress (Kausar et al., 2022; Kosova et al., 2021, Kosova et al., 2018; Nawae et al., 2020). Amino acids are key molecules for plant growth and development and in response to abiotic stress. Among them, asparagine (*Asn*) has the highest expression level in plants and plays a crucial role in plant stress tolerance. *Asn* is involved in multiple processes in plants, such as nitrogen metabolism, protein synthesis, and storage (Iqbal et al., 2022). Under non-biological stress conditions, including drought stress, plants accumulate *Asn* as a compatible solute (Altman, 2003; Qu et al., 2019), Therefore, further studying the role of *Asn* gene in plant abiotic stress response is of great significance for improving plant stress tolerance and sustainable agricultural production. Under drought stress, ASN and SNP treatment enhances drought resilience in plants by increasing osmolyte concentration, relative water content, and leaf water potential, promoting osmolyte synthesis. Our research shows that *A. mongolicum* resists drought stress by accumulating more *Asn*. Akin et al (Akin and Kaya, 2024). research found that the synergistic effect of *Asn* and *SNP* on osmolyte synthesis and water status is more pronounced, suggesting their potential as a complementary strategy for improving plant drought resistance. The stronger synergistic effect of *Asn* and *SNP* on osmolyte production and water status indicates their potential as a complimentary approach to enhancing plant drought resistance. It is likely that *Asn* and NO interact with each other and the plant in certain ways that increase the plant’s tolerance to drought, which might account for the synergistic action of these two compounds in enhancing the water relations of cotton plants during drought stress. *Asn* plays a role in osmoregulation and supports the maintenance of cellular turgor under drought stress (Schwendner et al., 2018; Yadav et al., 2019).

A variety of plant species harness the key function of *Asn* in response to abiotic stress. Under stress conditions, increased activities of asparagine synthetase (*AS*) and glutamate dehydrogenase help counteract NH^4+^ accumulation. Asparagine (*Asn*), a product of *AS*, plays a crucial role in nitrogen storage and transport due to its stability and high N:C ratio (Lam et al., 1996). In energy-limited conditions such as salinity, where glutamine synthetase (GS) and glutamate synthase (GOGAT) are inhibited and *AS* is activated, *Asn* is involved in nitrogen recycling and flow in plant cells, promoting nitrogen assimilation into *Asn*, which is rich in nitrogen and suitable for long-distance transport or long-term storage. Consequently, the upregulation of *OsAS1* enhances salt tolerance and grain yield in rice under salt stress conditions. Furthermore, *Asn*, the main N-transporter in alfalfa, accumulates in nodules and has been associated with N feedback, which inhibits symbiotic N_2_ fixation (King and Purcell, 2005; Larrainzar et al., 2009; Serraj et al., 2001). Studies have shown lower transpiration may alter the long-distance transport of nitrogen compounds, leading to an accumulation of nitrogen compounds in nodules under water stress. However, recent studies have not found any accumulation of nitrogen compounds in pea nodules under artificial reduction of plant transpiration, indicating that transpiration affects the long-distance transport of metabolites and its effects on the drought-induced inhibition of symbiotic nitrogen fixation (Aldasoro et al., 2019). In our study, we found that under drought stress, the accumulation of *Asn* in *A. mongolicum* significantly increased and the abundance of *Asn* is about five times that of other genes, playing an important regulatory role in the biosynthesis metabolism pathway of amino acids. *Asn* can participate in regulating plants’ responses to drought stress, helping plants better adapt to drought environments.

Moreover, the variable number of genes encoding *ASNS* among plant species, such as the single gene identified in alfalfa (Shi et al., 1997), rice (Nakano et al., 2000), soybean (Yamagata et al., 1998), and asparagus (Davies and King, 1993). Multiple *ASNS* genes have been found in pea (Tsai and Coruzzi, 1990),sunflower (Herrera-Rodríguez et al., 2002, Herrera-Rodríguez et al., 2004), soybean (Hughes et al., 1997), barley (Moller et al., 2003) and *Arabidopsis*. Some *ASNS* genes (e.g. pea *AS1*, *AtASN1*, sunflower *HAS1*, *HAS1.1*) are negatively regulated by light and sugars, being primarily expressed in dark-grown plants (Herrera-Rodríguez et al., 2004). RNA profiling of wheat identified three putative *ASNS* genes that were up-regulated in response to drought stress (Mohammadi et al., 2007). The result is the same as the experimental results. Under severe drought conditions, *ASNS*, which accumulates in ageing leaves, suggesting drought-induced senescence (Begona Herrera-Rodriguez et al., 2006; Eason et al., 2000). In a recent study, metabolites belonging to the aspartate pathway (including *Asn*, Ser, and Met) were reported as biomarkers for yield gap-based drought tolerance, accurately predicting more than 94% of drought tolerance in wheat (Yadav et al., 2019).

Drought responses in plants are closely related to the enzymes *P5CS* and *ProDH* involved in proline metabolism, which are influenced by soil moisture. H_2_O_2_production is also related to soil moisture across various stages of development, emphasizing the importance of considering seedlings and soil moisture conditions when studying drought stress. These results highlight the significant influence of soil moisture treatment on plant drought stress and provide insights into the underlying mechanisms. H_2_O_2_, as a ROS, plays a crucial role in intracellular communication and participates in plant adaptation to specific conditions. Oxidative stress caused by unfavorable environmental conditions, such as heat or drought, results in excessive ROS accumulation and triggers protective mechanisms, including proline accumulation through the upregulation of *P5CS* activity and the decrease of *ProDH* activity. Thus, H_2_O_2_ is involved in proline metabolism as a regulatory signaling molecule (Ben Rejeb et al., 2014; Uchida et al., 2002; Wen et al., 2013; Yang et al., 2009). This conclusion is consistent with the experimental results.

Drought resistance mechanisms in plants are diverse, with increasing root length and osmotic pressure regulatory substances being common strategies. as a crucial osmotic regulatory substance, proline plays a pivotal role in lowering the water potential of plant cells, strengthening their water absorption and retention capabilities, and thus safeguarding cellular osmotic balance and subcellular structural stability (Trovato et al., 2008). *P5CS* genes, a key role in plant proline biosynthesis, have been reported in a variety of plant species, such as A. thaliana, Cajanus cajan, N. benthamiana, and Oryza sativa (Guo et al., 2024; Igarashi et al., 1997; Ku et al., 2011; Su and Wu, 2004; Surekha et al., 2014) P5CS, a key enzyme in the proline biosynthesis pathway, not only enhances root growth but also plays a crucial role in plant resistance to drought. The electrical conductivity of cell leakage provides an indirect assessment of cell membrane damage (Bajji et al., 2002). *OE-SpP5CS* in *A. thaliana* exhibited a lower degree of membrane damage compared to the wild type (WT). Transgenic rice with *P5CS* increased their proline content under water shortage conditions and demonstrated an increased fresh shoot weight of 50-95% after PEG treatments (Su and Wu, 2004). The overexpression of *SpP5CS* in *A. thaliana* augments the proline content to enhance drought stress resistance. Drawing upon the results from heterologous expression in A. thaliana and qPCR in S. purpurea, it can be concluded that SpP5CS enhances the drought resistance of plants by boosting proline production.

*LKR* is a key enzyme in the lysine metabolism pathway, involved in the conversion of lysine to alpha-amino hexanoic acid, which is part of the saccharopine pathway. Lin et al (Lin et al., 2023). study identified proline as a crucial metabolite in grapevines’ responses to water stress. The study also discovered that the expression levels of genes associated with proline synthesis, such as *P5CS* and *LKR/SDH*, are upregulated during drought stress. Furthermore, the research suggests that grapevines bolster their drought resistance through the production of proline, a process mediated by *P5CS* and *LKR/SDH* enzymes (Degu et al., 2019; Hochberg et al., 2013)

### 4.2 Auxiliary pentose phosphate pathway under drought stress

In this study, several genes screened are involved in the glycolysis and pentose phosphate pathways. The pentose phosphate pathway (PPP) is closely linked to glycolysis and contributes to numerous metabolic pathways, contributing to numerous metabolic pathways. Sugars are vital participants in alleviating plant tolerance to abiotic stress (Keunen et al., 2013). Carbohydrate metabolism serves as a primary pathway in regulating cellular carbon and energy demands during drought stress, particularly through the accumulation of water-soluble sugars, which is of great significance for the induction of drought resistance in rice. Research on PEG-induced drought responses have demonstrated alterations in carbohydrate metabolism across various plant species, including lilac, pitaya, lentil, soybean, perennial ryegrass, alfalfa, sorghum and tomato (Abdel-Ghany et al., 2020; Cao et al., 2018; Foti et al., 2021; Guo et al., 2020a; Li et al., 2015; Siddiqui et al., 2021; Wang et al., 2021; Zhang and Shi, 2018; Zhou et al., 2022).

As knows *ALDH7B4* is primarily enriched in the pentose phosphate pathway. Expression of plant *ALDH7B4* is responsive to turgor and can protect cells from oxidative stress. In *Arabidopsis thaliana*, the *ALDH7B4* protein is induced by ABA, osmotic, and wound stress, as well as transgenic overexpression, leading to osmotic and oxidative stress tolerance (Hou and Bartels, 2015). Ectopic expression of the soybean *ALDH7* gene in tobacco and *A.thaliana* reduces MDA levels and sensitivity to hydrogen peroxide and methyl viologen, while rice *ALDH7 T-DNA* insertion mutants show increased sensitivity to various stresses (Kirch et al., 2004; Kotchoni et al., 2006; Missihoun et al., 2014). These findings collectively indicate that *ALDH7B4* plays a crucial role in plant adaptation and tolerance to abiotic stress (Shin et al., 2009; Suzuki et al., 2016).

## 5 Conclusion

This study conducted an analysis of transcriptome, proteome, and metabolome data from the shoots and roots of *A. mongolicum* seedlings to elucidate the underlying mechanisms of drought response and tolerance. The study found that arginine, proline, and Pentose Phosphate pathway metabolites play significant roles in the drought resistance of A. mongolicum. The comprehensive analysis of the integrated data revealed a synchronized response of genes, proteins, and metabolites associated with amino acid and pentose phosphate metabolic pathways in A. mongolicum under drought stress. This integrated approach is expected to enhance detailed investigations into the mechanisms of abiotic stress tolerance and response in A. mongolicum and potentially in other plant species.

## Data Availability

The original contributions presented in the study are included in the article/**Supplementary Material**. Further inquiries can be directed to the corresponding authors.

## Supplementary material

Supplementary Figure 1 After 24 hours of drought treatment, the proline content in the seedlings of *A. mongolicum* significantly increased.

Supplementary Figure 2 Protein level identification results.

Supplementary Figure 3 Under drought conditions, The KEGG pathway maps for Arginine and proline metabolism(left) and Pentose phosphate metabolism(right).

Supplementary Figure 4 Expression analysis of ASNS3, P5CS1 LKR and ALDH7B4 genes during drought treatment ing to qRT-PCR.

Supplementary Figure 5 correlation-network diagram.
