# Supplementary material for: Integrated transcriptomic, proteomic and metabolomic analyses revealing the roles of amino acid and sucrose metabolism in augmenting drought tolerance in Agropyron mongolicum
Source: Front Plant Sci. 2024 Dec 16;15:1515944. doi: 10.3389/fpls.2024.1515944 (PMC11685866; doi:10.3389/fpls.2024.1515944)
Supplement: Supplementary Figure 1 — After 24 hours of drought treatment, the proline content in the seedlings of A. mongolicum significantly increased. [file Table11.docx]

**Supplementary figures**

**Supplementary figure 1. After 24 hours of drought treatment, the proline content in the seedlings of *A. mongolicum* significantly increased.** Data are means ± SD (n = 3), “****” indicates a statistically significant difference at P < 0.0001, “ns” indicates no significant.

**
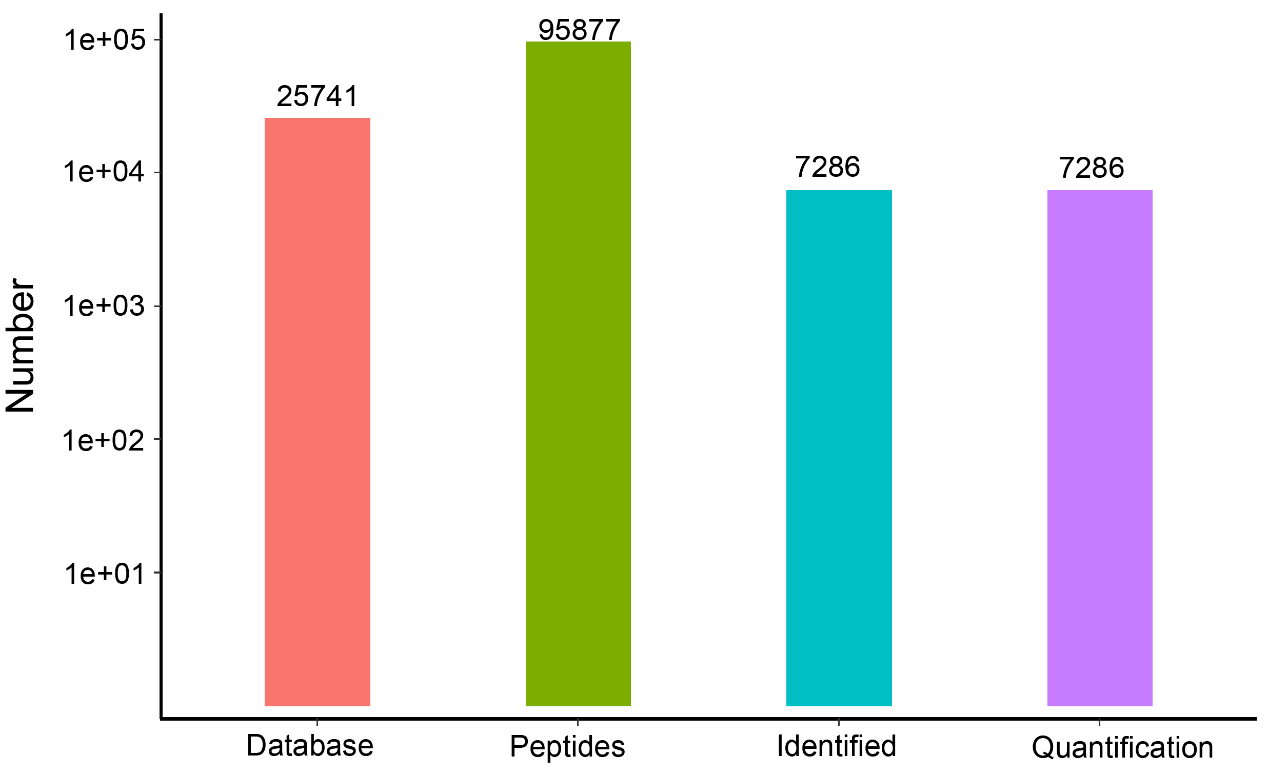
**

**Supplementary figure 2. Protein level identification results**

**
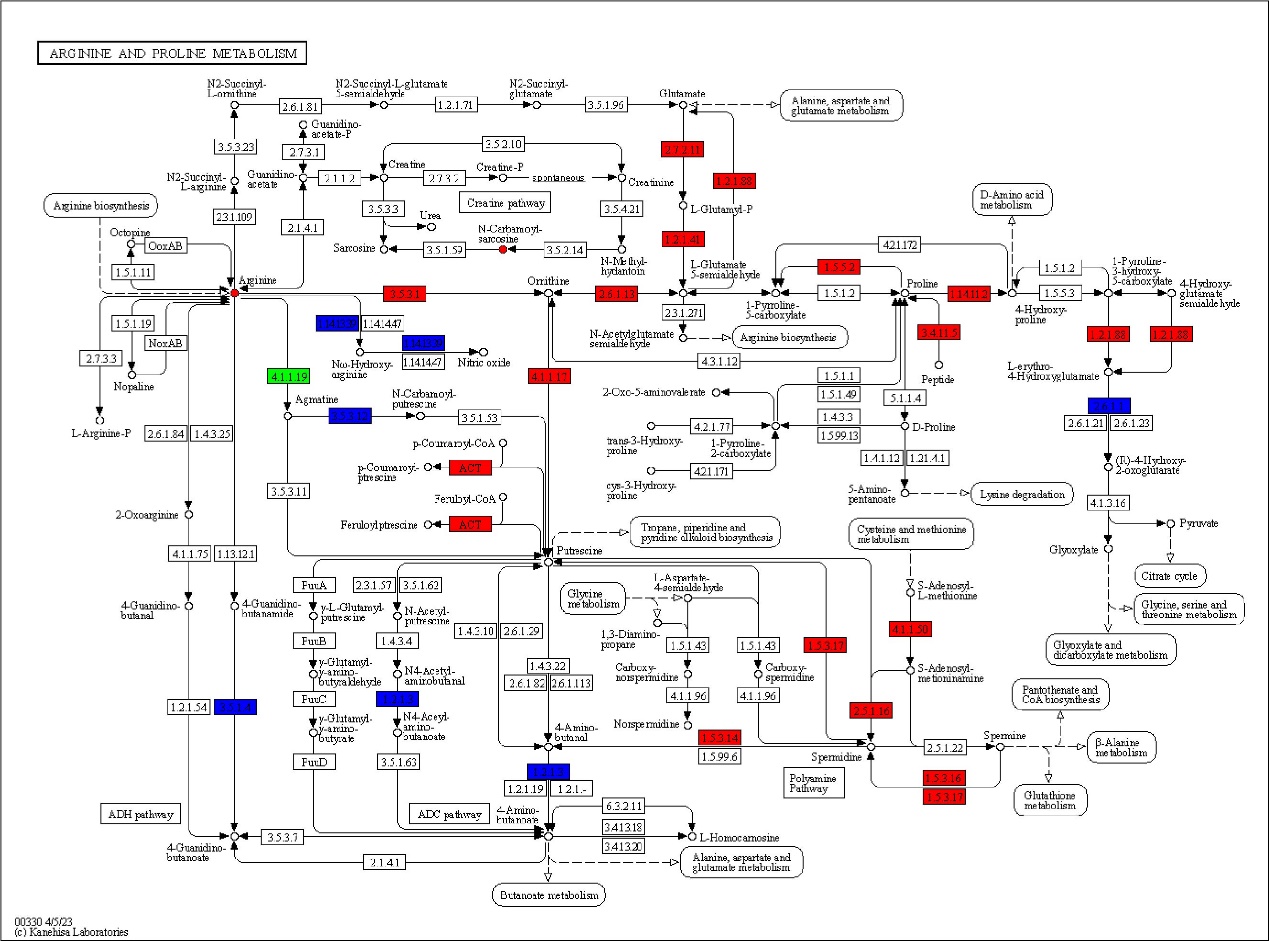
**

**
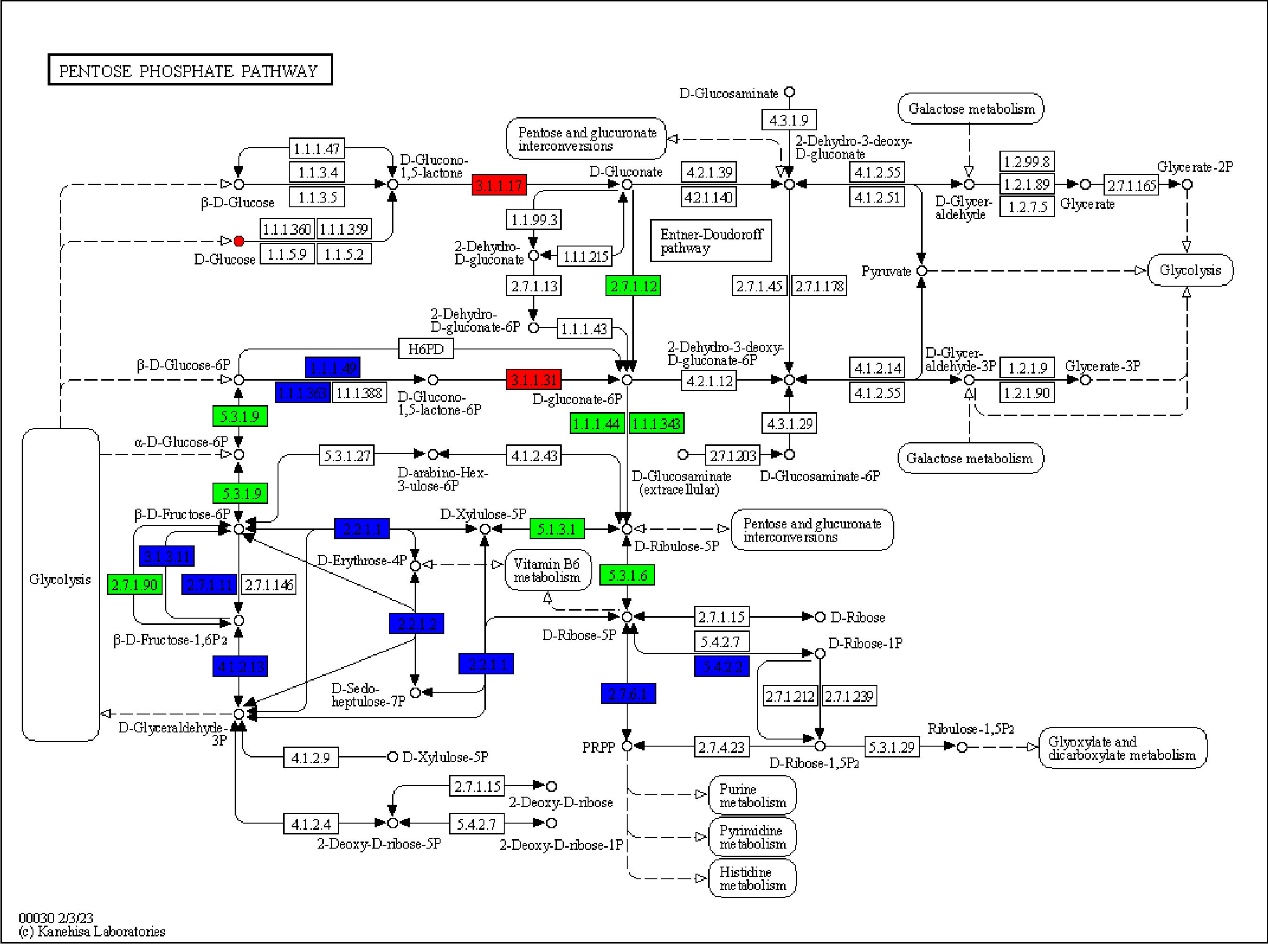
**

**Supplementary figure 3. Under drought conditions, The KEGG pathway maps for Arginine and proline metabolism(left) and Pentose phosphate metabolism(right).** small circles represent metabolites, and squares represent genes or proteins. Red indicates the upregulation state of genes, proteins, or metabolites, while green indicates the downregulation state. Blue represents genes or proteins that contain both upregulated and downregulated states.

**
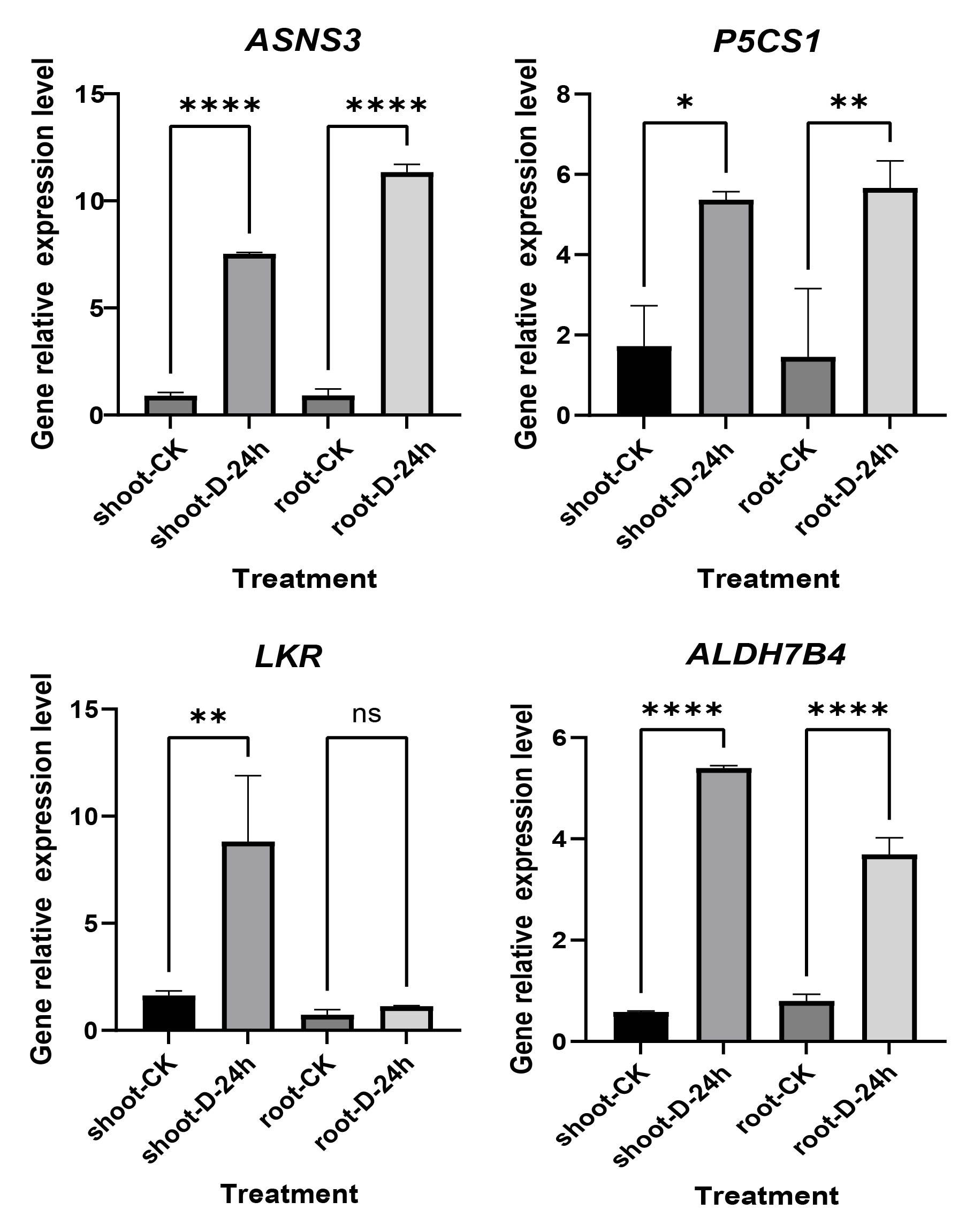
**

**Supplementary figure 4. Expression analysis of ASNS3, P5CS1 LKR and ALDH7B4 genes during cold treatment according to qRT-PCR.** Data are means ± SD (n = 3). Data are means ± SD (n = 3), “*” indicates a significant difference at P < 0.05, “**”indicates a statistically significant difference at P < 0.01, “****” indicates a statistically significant difference at P < 0.0001, “ns” indicates no significant.


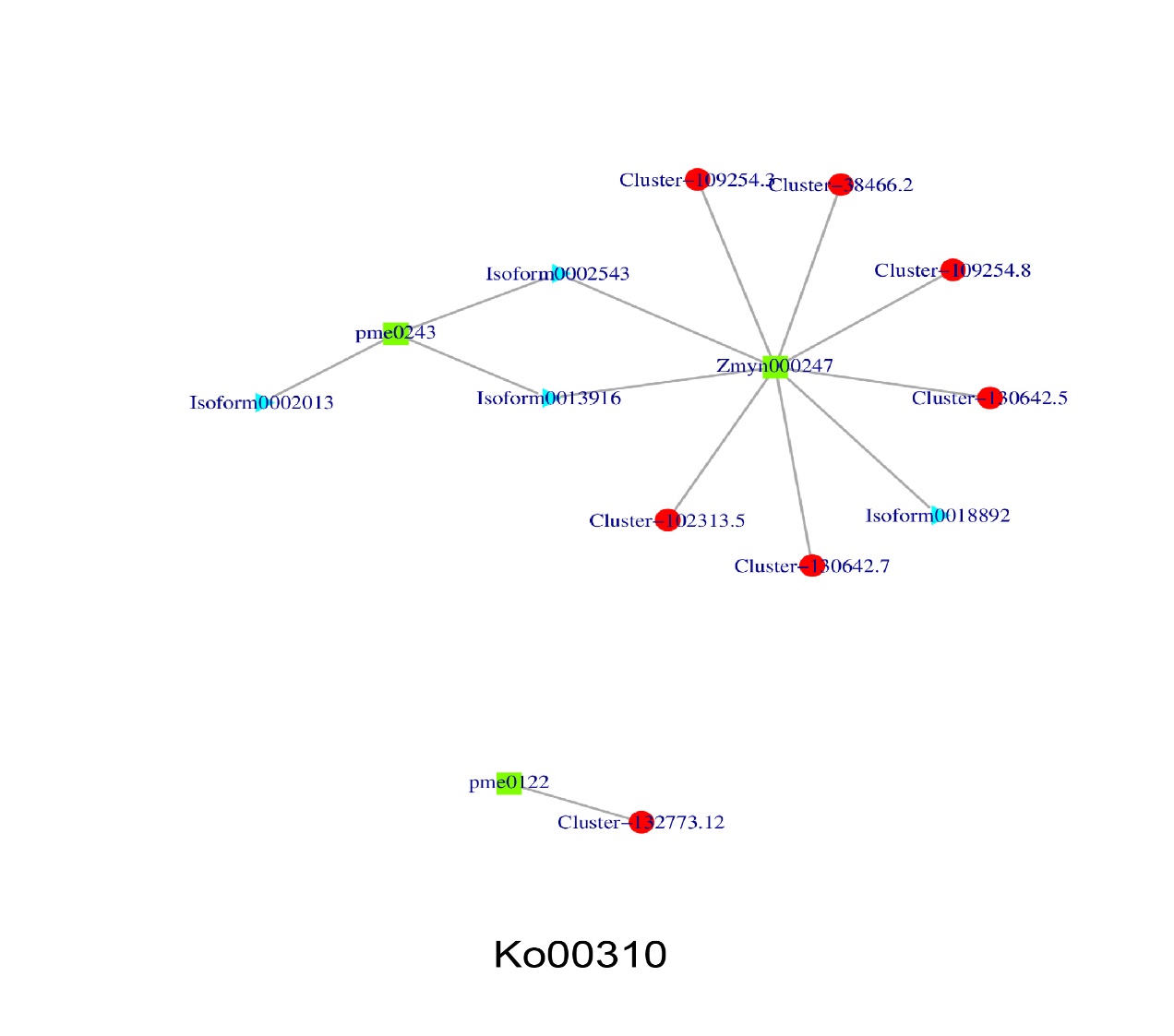

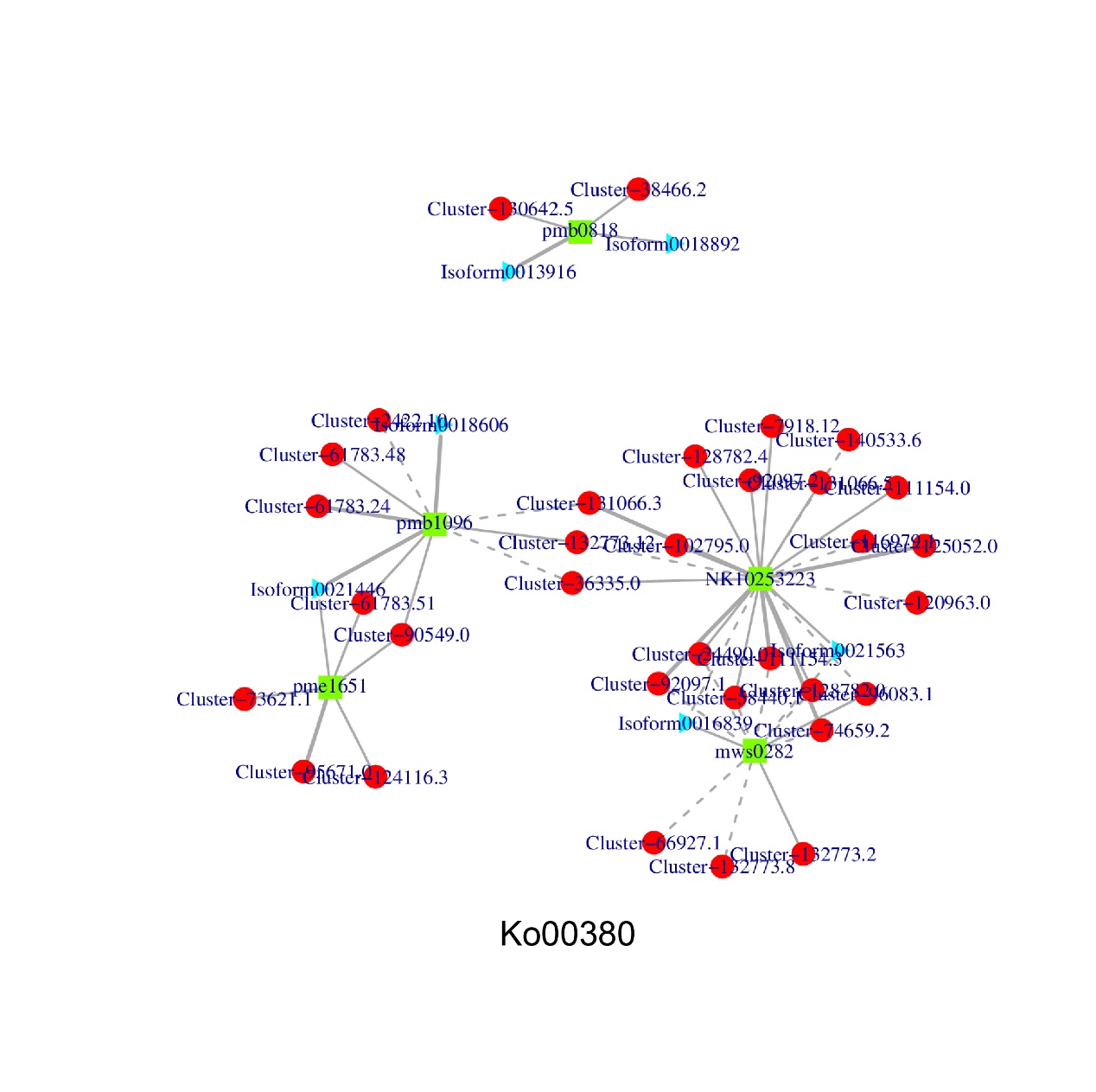

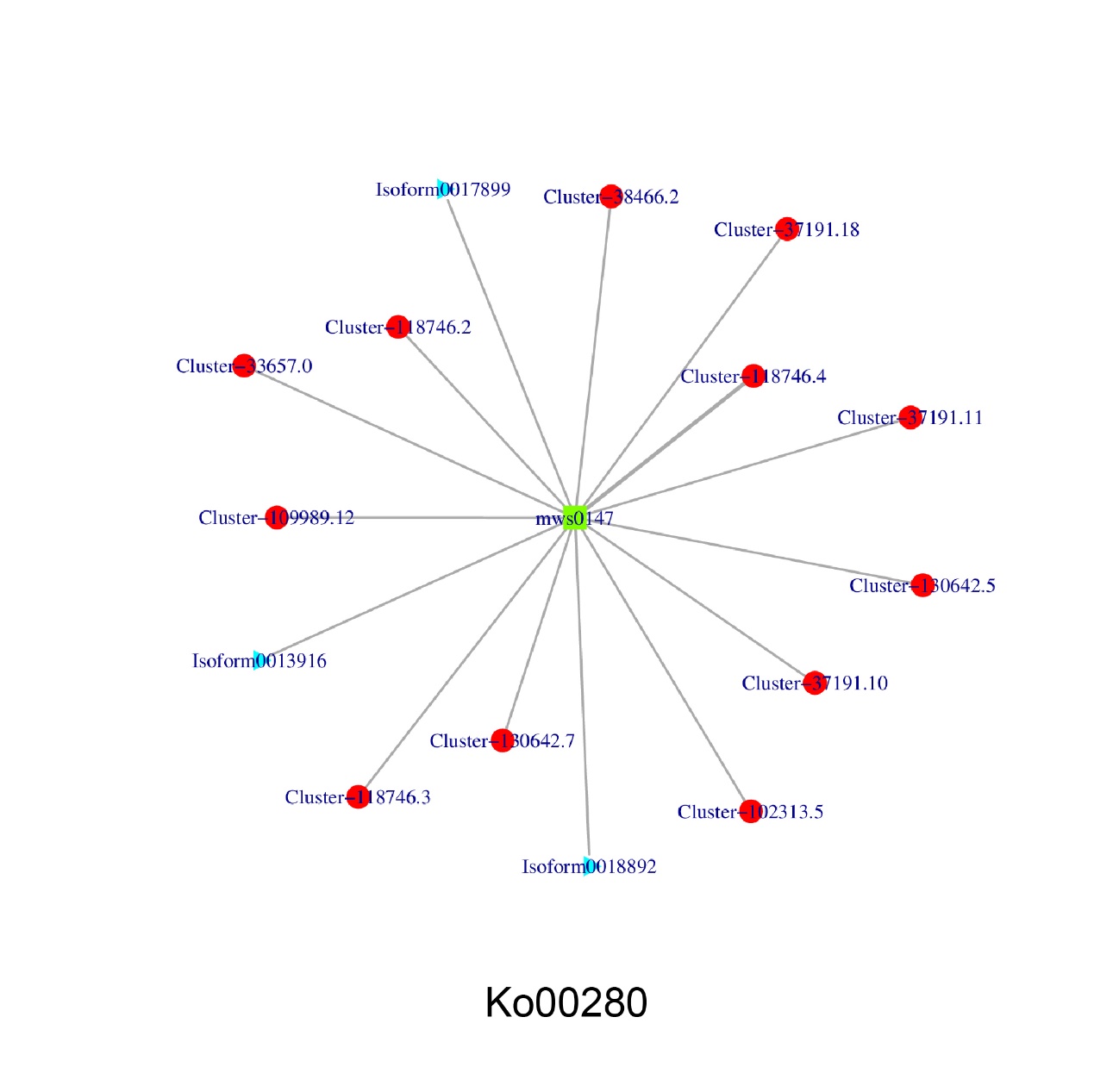


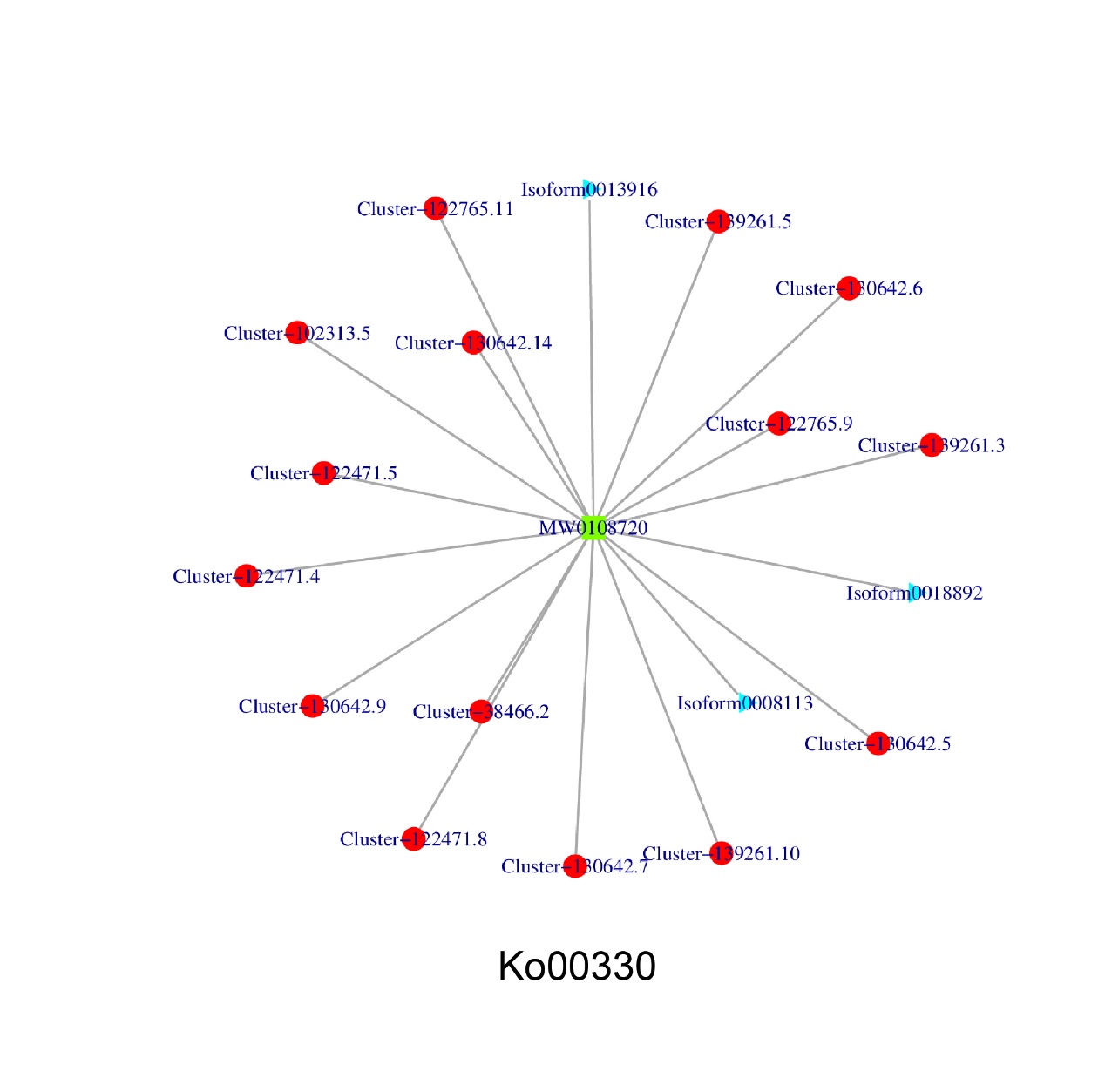


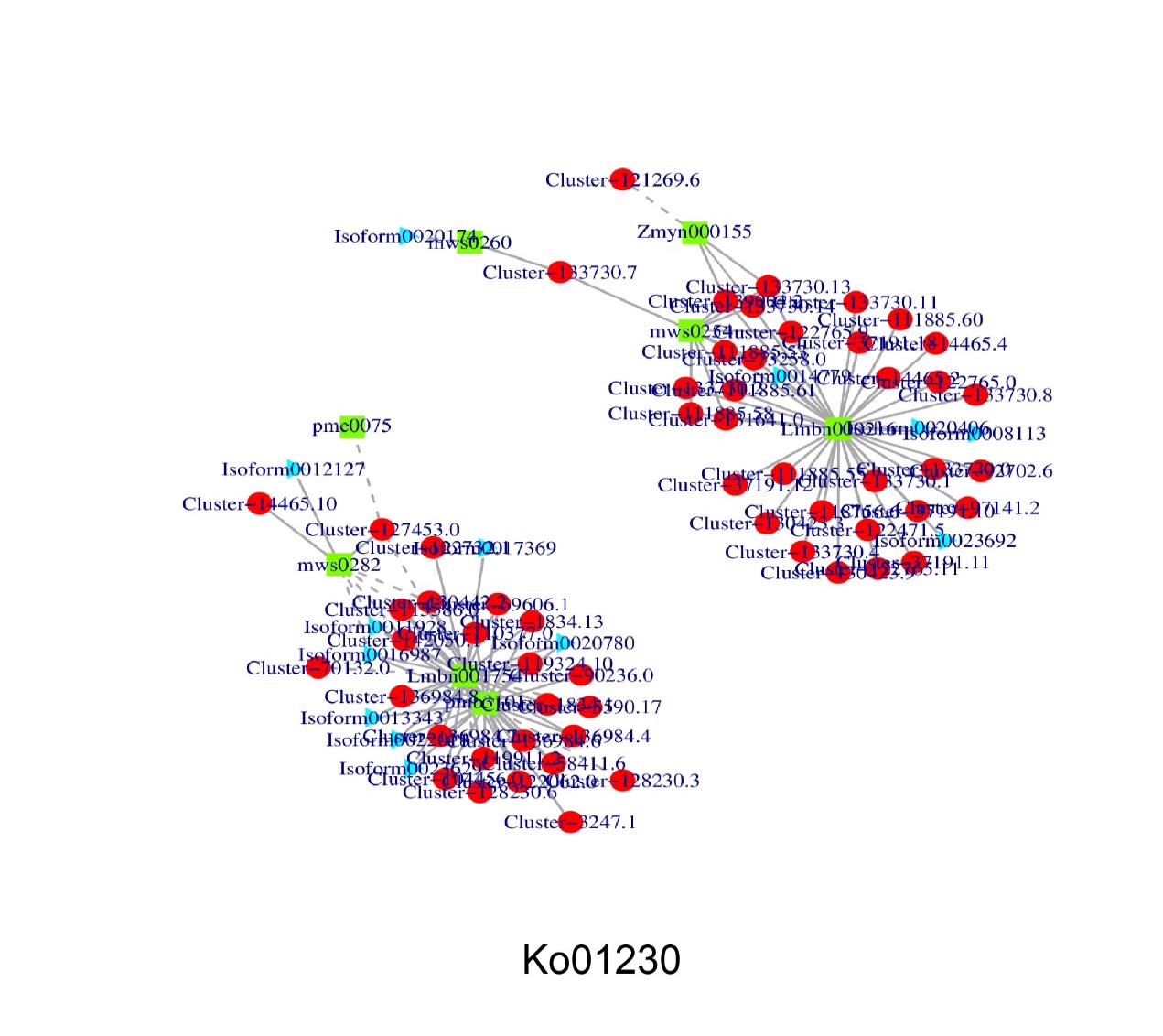


**
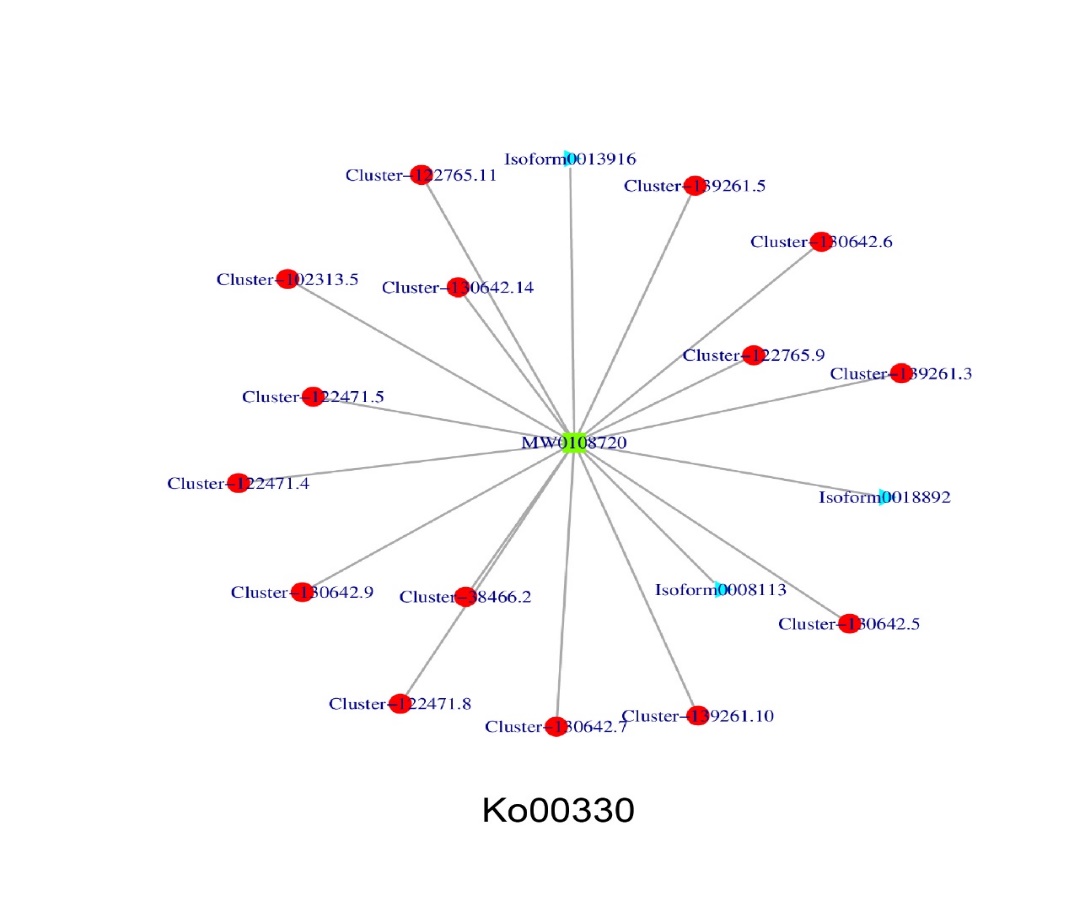
 Supplementary figure 5 correlation-network diagram**
